# The *RabGAP* Gene Family in Tomato (*Solanum lycopersicum*) and Wild Relatives: Identification, Interaction Networks, and Transcriptional Analysis during Plant Development and in Response to Salt Stress

**DOI:** 10.3390/genes10090638

**Published:** 2019-08-23

**Authors:** José Madrid-Espinoza, Josselyn Salinas-Cornejo, Simón Ruiz-Lara

**Affiliations:** Laboratorio de Genómica Funcional, Instituto de Ciencias Biológicas, Universidad de Talca, Talca 3460000, Chile

**Keywords:** RabGAP, vesicular trafficking, genome wide identification, evolution, salt stress response, tomato

## Abstract

RabGTPase activating proteins (RabGAP) are responsible for directing the deactivation of vesicular trafficking master regulators associated to plant development, the RabGTPase proteins. Recently, RabGAPs were identified in Arabidopsis and rice, but studies were not yet reported in tomato. Herein, we identified 24 RabGAP-encoding genes in cultivated tomato (*Solanum lycopersicum*) and its wild relative genomes (*Solanum pimpinellifolium* and *Solanum pennellii*). We analyzed them based on their exon-intron structures, conserved protein motifs, putative subcellular localizations, phylogenetic and gene duplications analyses, interaction networks, and gene expression patterns in tomato. Phylogenetic relationship analysis also indicated that RabGAP family is classified into seven subclasses, of which subclasses I and II are plant-exclusive. Furthermore, segmental duplication events and positive evolutionary forces are associated with the maintenance of the number and function of their members. On the other hand, the protein–protein interaction networks on tomato suggested that members of subclasses I, II, and III could be associated to endocytic traffic routes. In addition, the qRT-PCR experiments in *S. lycopersicum* and *Solanum chilense* exposed to a salt stress treatment validated the differential expression patterns of 20 RabGAP genes in different tissues, development stages, and stress conditions obtained through extensive microarray-based analyses. This work suggests the critical role of RabGAP family in the context of intracellular vesicular trafficking in tomato, particularly under conditions of abiotic stress. It also contributes to the breeding programs associated with the development of crops tolerant to salt stress.

## 1. Introduction

Abiotic stress represents the main environmental challenge in the production of agronomically important crops. Salt, drought, or heat, among other stresses, alter the internal homeostasis of plants, damaging their tissues and organs, as well as reducing their yield [1]. To counteract these negative effects, mechanisms for synthesis and accumulation of compatible osmolytes [2], detoxification of reactive oxygen species [3], mobilization of ion and water transporters [4,5], or synthesis and mobilization of lipids are activated [6]. The performance of these mechanisms depends on the efficient vesicular traffic between the different subcellular compartments [7,8,9]. The small GTPases of the Rab family (RabGTPases) regulate the vesicular traffic, alternating between an “active” state GTP-bound and an “inactive” state GDP-bound, as a molecular switch. The activated state is dependent on guanine nucleotide exchange factors (GEF) proteins, which facilitate the GDP dissociation by GTP [10]. Then, RabGTPase proteins are recognized by effector proteins such as homotypic fusion and protein sorting (HOPS), transport protein particle (TRAPP), and class C core vacuole/endosome tethering (CORVET) complexes, among others, helping membrane fusions [11]. The “inactive” state of RabGTPase is promoted by association with RabGAP proteins. The RabGAP proteins hydrolyze GTP, allowing RabGTPases to be bio-available for a new cycle of vesicular trafficking. In plants, each RabGTPase directs a particular traffic route, but the knowledge of the routes in which many RabGAP proteins participate is still very limited [12].

Unlike yeast and animals, little is known about the RabGAP proteins in plants, the mechanisms that they modulate, their transcriptional regulation, or their role in the stress tolerance. From a genomic analysis in yeast, all RabGAPs should have a conserved TBC-like domain (Tre-2/Bub2/Cdc16) with a hydrolytic activity directed by an arginine and glutamine that act as a dual-finger mechanism on GTP-bound RabGTPases [13]. Other domains associated with RabGAP proteins are the Rab3-GTPase_cat and Rab3GAP2_N domains, which are specific for RabGTPase proteins of subfamily D (RabD) [14]. In plants, 25 and 24 RabGAP genes have been identified in rice and *Arabidopsis thaliana*, respectively [15], whereas in *Vitis vinifera*, only five RabGAPs have been found by EST analysis [16]. In general, the RabGAP family has been subdivided into nine subclasses (I to IX), of which the subclasses III, IV, V, VI, and VIII are present in plants, yeast, insects, and animals, whereas the subclasses I and II are plant-specific [15].

During environmental stress the vesicular trafficking mechanisms are up-regulated. For example, RabGTPase genes are regulated in a positive way, increasing the endocytosis, the vesicular traffic, and the vacuolar compartmentation of ions [9]. Among them, Rab5 of *Mesembryanthemum crystallinum*, a salt tolerant species, or OsRab7 of rice, increase their expression during in cold, dehydration or salt stress [17,18]. Other studies have demonstrated that the over-expression of RabGTPase genes associated to endocytosis mechanisms, like RabG3e and RabF1 in *A. thaliana* or PgRAB7 in tobacco, also increase the vacuolar compartmentalization of Na^+^ ions and the transport of proteins between organelles [9,19,20]. Likewise, the participation of proteins associated to molecular switching during abiotic stress has been evaluated. Among them, the overexpression of SchRabGDI1 of *Solanum chilense* is capable of increasing the endocytosis and salt tolerance of *A. thaliana* [21]. While the absence of AtVPS9, a GEF protein, compromises its salt stress tolerance [22]. However so far, the OsGAP1 protein from rice is the only RabGAP protein characterized in the context of abiotic stress [23]. Specifically, OsGAP1 interacts with RAB11, being both essential for vesicle trafficking of OsVHA-a1 (a vacuolar H^+^-ATPase) from trans-Golgi network (TGN) to the central vacuole and salt stress tolerance [24].

The cultivated tomato (*Solanum lycopersicum*) is one of the most important crops in the world. However, abiotic stress is the main limitation of their yield, development and agronomic properties [25]. On the other hand, wild tomato species such as *Solanum pimpinellifolium*, *Solanum habrochaites*, *Solanum pennellii*, and *S. chilense*, among others, are tolerant species to abiotic stress and thrive in extreme environments due to differential expression of a diverse set of genes that activate molecular and physiological mechanisms that allow them to effectively adapt [26,27]. The recent sequencing of the *S. pennellii*, *S. lycopersicum*, and *S. pimpinellifolium* genomes provides an excellent opportunity to identify and analyze the *RabGAP* gene family in the context of abiotic stress [28,29]. In this sense, we have identified each *RabGAP* gene from these tomato species, reporting amino-acid characteristics, phylogenetics, and the interaction analysis between RabGTPase–RabGAP proteins. Furthermore, through a comprehensive study of the transcriptional profiles in different tissues and stages of development, as well as during salt stress conditions, we provide a global overview of its putative biological and physiological functions. This research supplies an important framework for future research in tomatoes as well as for the development of genetic improvement strategies.

## 2. Materials and Methods

### 2.1. Identification and Phylogenetic Analysis of RabGAPs from Tomatoes

To determine the sequences corresponding to RabGAP proteins, a local database was established using the proteomes of *S. lycopersicum* (SlRabGAP), *S. pennellii* (SpeRabGAP), and *S. pimpinellifolium* (SpiRabGAP) obtained from the SolGenomics Network database (https://solgenomics.net) [30]. Here, the RabGAP proteins were determined by the specific TBC domain (Pfam PF00566) as a profile for Hidden Markov chains (HMM), with a cut-off value of 1e^−10^ in the HMMERv3.1 software [31]. Each protein was manually validated by comparative analyses with the Solgenomics, BLASTp (https://blast.ncbi.nlm.nih.gov), CDD (http://www.ncbi.nlm.nih.gov/Structure/cdd/wrpsb.cgi), and Pfam databases (http://pfam.xfam.org/) [32].

To assign a name to each RabGAP protein and determine the subclass to which it belongs, the complete length of the amino-acid sequence was aligned with its closest homolog of *A. thaliana* using the software ClustalO [15,33]. The phylogenetic tree was constructed with MEGA7 software using the neighbor-joining method with 5000 iterations bootstrap [34].

### 2.2. Sequence Amino-Acid Properties, Synteny and Gene Duplication Analysis

The isoelectric point (pI) and molecular weight (MW) were predicted with Expasy Compute pI/Mw tool (http://web.expasy.org/compute_pi/). The exon-intron structure of the genes was performed with the GSDS software (http://gsds.cbi.pku.edu.cn) [35]. In addition, the conserved motifs in the amino-acid sequence were identified using the web tool MEME (http://meme-suite.org) and analyzed with InterPro Scan 5 (https://www.ebi.ac.uk/interpro/) [36,37]. Values of hydropathy (GRAVY value) were calculated with the help of Expasy ProtParam tool (http://expasy.org/tools/protparam.html). The subcellular locations were determined using CELLO V2.5 (http://cello.life.nctu.edu.tw) [38], Wolf PSORT (https://wolfpsort.hgc.jp) [39], and MultiLoc2 tools (https://abi.inf.uni-tuebingen.de/Services/MultiLoc2) [40]. The predictions of protein-protein interactions were made with the STRING database (https://string-db.org) [41].

Synteny analysis was carried out using the chromosomal locations of homologous and paralogous *RabGAP* genes in *S. lycopersicum* and *A. thaliana* obtained from the PGDD database (http://chibba.agtec.uga.edu/duplication/) [42]. The graphic representation was made using the web-based service ClicO FS [43]. Along with this, the selection pressure of *RabGAP* genes was determined by the ratio of nonsynonymous to synonymous nucleotide substitutions (Ka/Ks) between paralogs in *S. lycopersicum*. The approximate date of the duplication events was estimated using T = (Ks/2λ) × 10^−6^ million years ago (mya), based on the clock-like rates (λ) in Solanaceae of 1.5 × 10^–8^ [44].

### 2.3. RabGAP Gene Expression Profiles in Databases

Tissue-specific expression profiles of the *RabGAP* genes of *S. lycopersicum* and *S. pennellii* were obtained from the Bio-Analytic Resource database from the University of Toronto (http://bar.utoronto.ca). The data were obtained from flowers, shoots, leaves, vegetative meristems, seedling shoots, seedling roots, mature fruit, and developing fruit [45]. To establish a relation between the expression profiles of the *RabGAP* genes and the capacity of tolerance to abiotic stress among *S. lycopersicum* and its wild relatives (*S. pimpinellifolium*, *S. habrochaites*, and *S. pennellii*), values normalized in respect to *S. lycopersicum* were obtained from the “Tomato Expression” database (http://malooflab.phytonetworks.org/apps/tomato-expression/) [45]. In addition, to study the expression profiles of *RabGAP* genes in response to abiotic stress, we used the PLEX database (http://www.plexdb.org). The data were extracted from L6 (GEO accession GSE16401), L12 and L13 experiments (GEO accession GSE22304). The L6 experiment corresponded to a salt stress assay considering samples of leaves from 6-week-old *S. lycopersicum* and *S. pimpinellifolium* treated with NaCl 200 mM for 5 h [46]. The L12 experiment correspond to drought stress considering samples of leaves from four-week-old *S. lycopersicum* and *S. habrochaites*, previously non-irrigated for 14 days. Finally, the L13 experiment corresponded to a heat stress assay considering samples of leaves from four-week-old *S. lycopersicum* and *S. habrochaites* treated at 40 °C [47]. The profiles are relative to the control without stress.

### 2.4. Plant Material and Gene Expression Analysis

Seeds of *S. chilense* (Dunal) and *S. lycopersicum* were germinated in a mixture of perlite, vermiculite and peat moss (1:1:1), grown under greenhouse conditions at 23–25 ºC, with photoperiod of 16/8 h light/dark, irrigated with 400 mL every five days and fertilized with a commercial Hoagland’s solution (1/4 strength) every 10 days. At the sixth week, a saline stress of NaCl 300 mM was applied to a group of plants. Leaves and roots were collected at 0, 3, 6, 12, 24, 48, and 72 h for the gene expression analyses. Then, total RNA from both tissues of *S. chilense* was extracted, treated, and quantified following the protocol of San Martín-Davison et al. (2017) [21]. To obtain the first strand of cDNA, 2 μg of RNA was retro-transcribed in 20 μL of reaction using the First Strand cDNA Synthesis Kit (Thermo Scientific, Waltham, MA, USA) and following the manufacturer’s instructions.

Relative levels of gene expression were measured by qRT-PCR, using the Stratagene Mx3000p thermocycler (Agilent Technologies, Santa Clara, CA, USA). Maxima SYBR Green/ROX qRT-PCR Master MIX (Thermo Scientific) was used for all reactions according to the protocol described by the manufacturer. For each sample (three biological replicates), qRT-PCR was performed in triplicate (technical replicates) using 10 μL of Master MIX, 0.5 μL of 250 nM of primers, 1 μL of cDNA and nuclease-free water in a final volume of 20 μL [48]. The amplification was followed by a melting curve with continuous fluorescence measurement at 55 to 95 °C. The data were manually analyzed, and the expression normalized with the *Ubiquitin3* gene [49]. All primers are listed in Table S1. The transcript levels of each gene were evaluated using the 2^−ΔΔCT^ method [50]. The qRT-PCR data were analyzed using ANOVA (one-way analysis of variance) tests (with a significance of *p*-value < 0.05). Data management and standardization were performed with R version 3.2.5 [51].

## 3. Results

### 3.1. 24 Proteins Organized in 7 Subclasses Constitute the RabGAP Family in Tomato

An analysis of HMM on the proteomes of *S. lycopersicum*, *S. pimpinellifolium*, and *S. pennellii* allowed us to identify 24 putative RabGAP proteins in each species. They were named according to the closest ortholog of *A. thaliana* [15]. Among them, 21 RabGAPs possessed the TBC domain, two the Rab3-GTPase_cat domain and one the Rab3GAP2_N domain [14,52]. At the amino-acid level, all RabGAP proteins had the conserved residues of arginine and glutamine, critical for catalytic activity. The exceptions were RabGAP23b and those with the Rab3-GTPase_cat and Rab3GAP2_N domains, see in Figure S1. In *S. lycopersicum*, the RabGAP proteins had a length between 352 (SlRabGAP18) and 942 amino-acids (SlRab3GAP2) and a theoretical isoelectric point ranging from 4.79 (SlRabGAP6) to 8.97 (SlRabGAP18). Similar characteristics were also observed in the RabGAP protein families of *S. pennellii* and *S. pimpinellifolium*. On the other hand, the GRAVY index, associated to the hydrophobicity level of a protein, was less than 0, revealing that all RabGAPs are hydrophilic. In this same sense, the prediction of subcellular localization using three different web servers indicated that the main locations for all RabGAP proteins were in the nucleus and cytoplasm, as shown as in Table 1.

**Figure 1:**
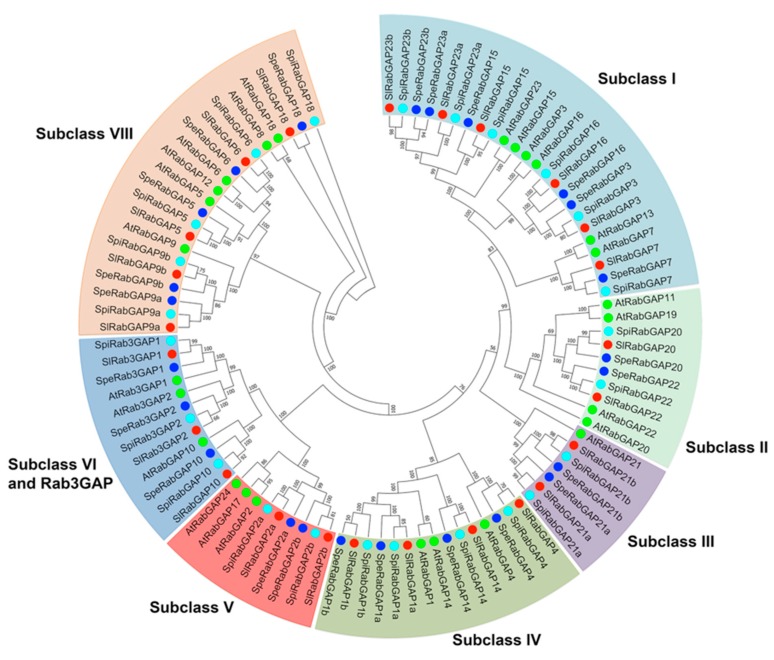
Phylogenetic tree of tomato RabGAPs. Seven subclasses (I, II, III, IV, V, VI, and VIII) were identified. 26 RabGAP of *A. thaliana* [15] are represented in green circles. In red, 24 RabGAP of *S. lycopersicum*; in light blue, 24 RabGAP of *S. pimpinellifolium*; and in dark blue, 24 RabGAP of *S. pennellii*.

In respect to the classification of RabGAP proteins, the phylogenetic analysis evidenced that they are grouped in seven subclasses (I, II, III, IV, V, VI-Rab3GAP, and VIII), according to their homologs in *A. thaliana*, shown in Figure 1 [15]. This analysis revealed that the subclass with the most members was subclass I and those with the fewest members were subclasses III and V. In addition, subclasses II, V, and VIII of *S. lycopersicum* had fewer members than those of *A. thaliana*, while subclasses III and IV had more.

### 3.2. Events of Segmental Duplication Have Kept Constant the Number of Members of the RabGAP Family

**Figure 2:**
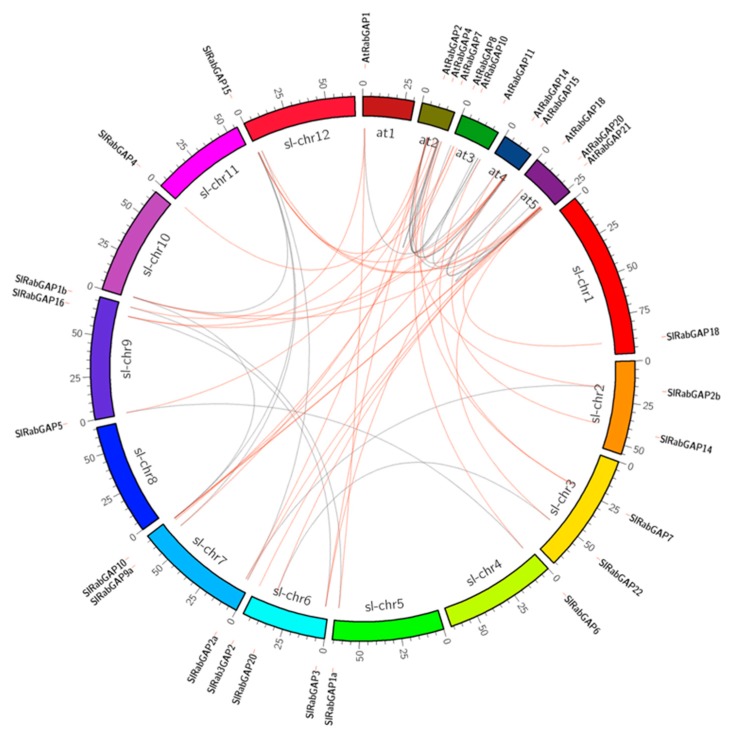
Syntenic relationships among RabGAP. Paralogy and orthology relations between RabGAP from *S. lycopersicum* and *Arabidopsis thaliana*. Orthologous and paralogous genes are connected by red and gray lines, respectively.

To evidence the genomic organization and the role of duplication events in the evolutionary history of RabGAP family genes, synteny mapping, intron-exon structure identification and selection pressure analysis were performed, see in Figure 2. First, the genomic analysis revealed that the SlRabGAP genes were differentially distributed on 11 chromosomes of *S. lycopersicum*: One gene each on Sl-Chr1, -4, -5, -10, and -11; two genes each on Sl-Chr2 and Sl-Chr3; three genes each on Sl-Chr6, -9, and -12; and six genes on Sl-Chr7. Then, the comparative analysis between the exon-intron structures and their chromosome locations allowed us to identify seven events of segmental duplication (SlRabGAP20-SlRabGAP22, SlRabGAP21a-SlRabGAP21b, SlRabGAP1a-SlRabGAP1b, SlRabGAP9a-SlRabGAP9b, SlRabGAP5-SlRabGAP6, SlRabGAP2a-SlRabGAP2b, and SlRabGAP3-SlRabGAP16) and one triplication event between SlRabGAP23a, -23b, and -15. Along with the above, in all these cases the ratios of synonymous and non-synonymous nucleotide substitutions (Ka/Ks) were less than 1, indicating that the function of the genes has not diverged and has been maintained due to a stabilizing selection force. Finally, the estimated time in which these duplications occurred was 15 to 28 mya, see in Table 2.

**Figure 3:**
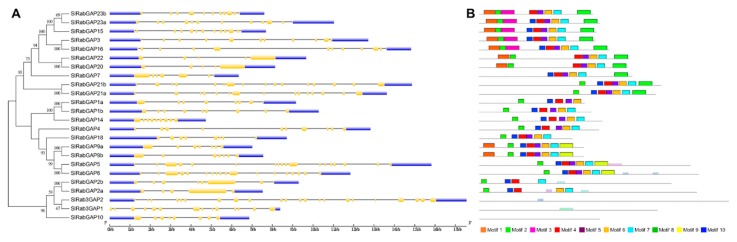
Identification of exon-intron structure and conserved motifs in RabGAP from *S. lycopersicum*. (**A**). Exon-intron structure of *RabGAP* genes from *S. lycopersicum*. In yellow, exons; in blue, the 3′ and 5′ UTR regions; and the gray lines are introns. (**B**) Conserved motif of RabGAP proteins. Ten motifs were identified with MEME database. The gray lines are the complete amino-acid sequence represented proportionally. Each color represents a different motif. Together, the pink, red, purple, orange, and light blue motifs represent the TBC domain. The consensus sequences are detailed in Table S2.

In relation to the characterization of the *RabGAP* gene structure, the results showed that the number of introns is very diverse, see in Figure 3, Figure 3A. On average, the RabGAP genes had 13 introns, of which RabGAP2a, -20, and -22 had the lowest number (four introns), whereas Rab3GAP2 had the highest number (21 introns). On the other hand, the RabGAP proteins were characterized by having between four or five conserved motifs associated with the “catalytic core” from the TBC domain, as shown in Figure 3, Figure 3B. Exceptionally, the catalytic domain of subclass VI, specifically associated with Rab3, was present in three proteins—Rab3GAP1, Rab3GAP2, and RabGAP10—as seen as in Table 1.

### 3.3. In Silico Analysis of Protein-Protein Interactions Suggests the Association of RabGAP Proteins with Specific Vesicular Trafficking Pathway

**Figure 4:**
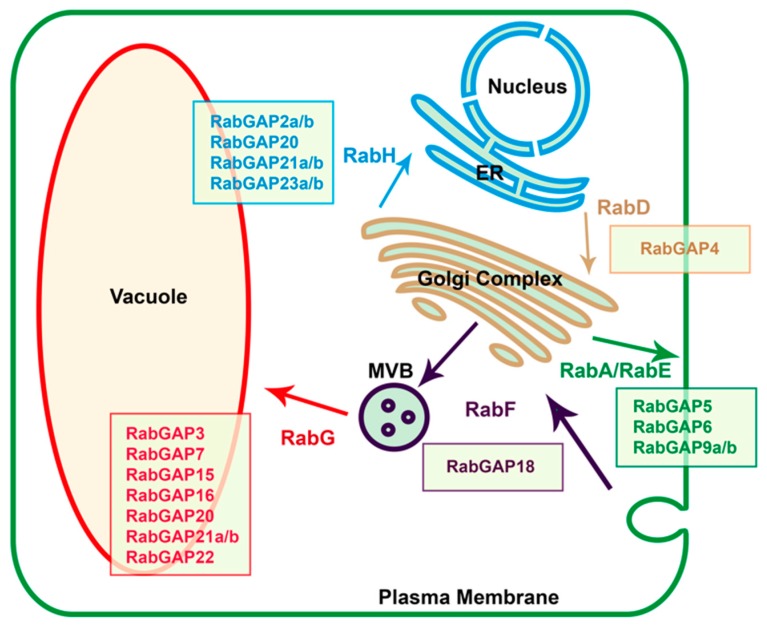
Identification of proposed protein-protein interactions between RABs and RabGAPs. The vesicular trafficking routes directed by the RabGTPase subfamilies between the different organelles and membranes are represented by arrows. RabGAP proteins were associated to each RabGTPase using the protein-protein interaction functional networks provided by the STRING database [41]. ER: Endoplasmic Reticulum. MVB: Multi-Vesicular Body. All proposed protein-protein interaction networks are detailed in Table S4.

In order to suggest specific vesicular trafficking pathways in which the RabGAP proteins could participate, we identified their possible affinities or physical interactions with the RabGTPase subfamilies of *S. lycopersicum* using the STRING database, see in Table S3. The results showed that in the exocytic pathway, SlRabGAP5, -6, -9a, and -9b could be interacting with RabGTPases of the subfamilies-A and E. In the anterograde pathway, RabGAP4 could be interacting with the subfamily-D, whereas in the retrograde pathway, SlRabGAP2a, -2b, -20, -21a, -21b, -23a, and -5 could be related with subfamily-H. In the endocytic pathway, only RabGAP18 could be interacting with subfamily-F, whereas SlRabGAP3, -7, -15, -16, -20, -21a, -21b, and -22 could be relating with subfamily-G (see in Figure 4).

We also observed that some RabGAPs could form protein complexes. Homodimers are formed in the case of SlRabGAP1a, -1b, -3, -4, -9a, -9b, -14, -16, -21a, -21b, or -23a; heterodimers between SlRabGAP10, -18 or -23a; protein complexes between vacuolar protein sorting (VPS) and SlRabGAP3, -7, -15, -20, -21a, -21b, -22, -23a, or -23b. The complexes between proteins related to cell division checkpoint processes, such as SlRabGAP5, -6, -7, -9a, or -9b, and complexes with proteins that respond to hormones involved in stress, such as SlRabGAP5 or -6, were also identified (see Table S2).

### 3.4. Differential Expression of RabGAP Genes in Different Tissues and Development Stages of Cultivated and Wild Tomato Species

**Figure 5:**
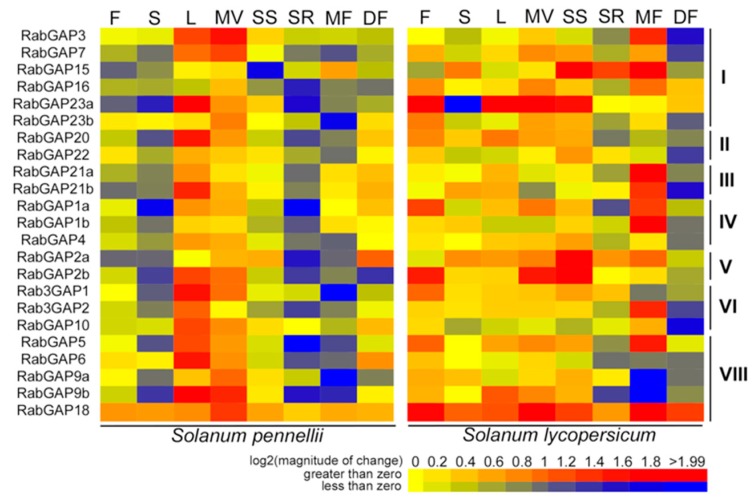
Expression patterns of RabGAP genes in different tissues, organs and developmental stages of tomato. Induction of genes is represented in red and repression in blue. F: flowers, S: shoots, L: leave, MV: vegetative meristems, SS: seedling shoots, SR: seedling roots, MF: mature fruit, DF: developing fruit. All values are detailed in Table S5.

To explore patterns in the transcriptional regulation of the RabGAP genes, we analyzed the expression profiles in different organs and development stages of *S. lycopersicum* and *S. pennellii* (see Figure 5, File S1). We evidenced that the expression patterns of RabGAP genes in flowers, shoots, seedling shoots, seedling roots, and mature fruit of *S. pennellii* were inverse to that of *S. lycopersicum*. Furthermore, in almost all organs of *S. lycopersicum* the expression patterns were positive, while in *S. pennellii*, they were only positive in leaves and vegetative meristems. At the subclass level, no obvious differences were observed. However, we highlight that the most up-regulated *RabGAP* genes in *S. lycopersicum* were SlRabGAP23a in flowers, leaves, and vegetative meristems, SlRabGAP18 in stems and developing fruit, SlRabGAP2a in seedling shoots, and RabGAP15 in seedling roots. While in *S. pennellii* were SpRabGAP18 in seedling shoots, seedling roots, flowers and stems. SpRabGAP23a in leave, SpRabGAP3 in vegetative meristems, SpRabGAP15 in mature fruit, and SpRabGAP2a in developing fruit. It is also interesting to mention that RabGAP18, the only RabGAP possibly associated with RabGTPase of the F subfamily, was ubiquitously expressed in all organs and stages of development of both wild and cultivated tomato.

**Figure 6:**
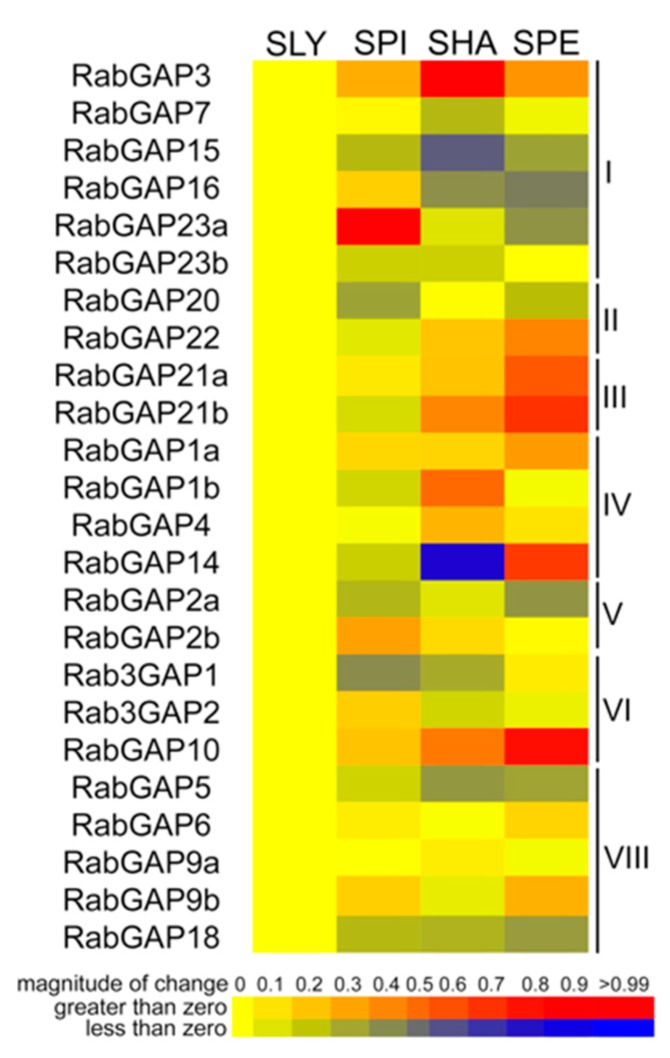
Expression profiling of RabGAP genes in wild tomato species. Transcript levels of RabGAP genes from wild tomato species relative to *S. lycopersicum* are represented. Higher or lower gene expressions are shown in red and dark blue, respectively. Nomenclature: SLY: *S. lycopersicum*, SPI: *S. pimpinellifolium*, SHA: *S. habrochaites* and SPE: *S. pennellii*. All values are detailed in Table S6.

To evidence differential patterns of regulation between tomato and wild relatives in normal conditions, we analyzed the expression profiles of the RabGAP genes in leaves of *S. pimpinellifolium*, *S. habrochaites*, and *S. pennellii*, see in Figure 6. SpiRabGAP23a, ShaRabGAP3, and SpeRabGAP10 were the most up-regulated genes in each wild species, while SpiRab3GAP1, ShaRabGAP14, and SpeRabGAP16 were the most down-regulated. Studying an expression pattern associated with the degree of tolerance to abiotic stress of each wild species, we observed that RabGAP1a, -10, and -21a had positive correlations, whereas only RabGAP18 had a negative tendency. In addition, the analysis showed that both RabGAP2a and -22 represented about 35% of the number of transcripts of RabGAP family (see Table S6).

### 3.5. RabGAP Genes Associated with Endocytic and Pre-Vacuolar Trafficking Are Up-Regulated in Roots Subjected to Salt Stress

**Figure 7:**
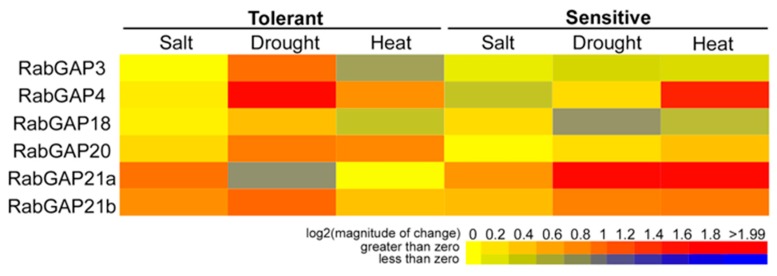
Transcriptomic profiles of RabGAP genes from tomato species with different degrees of abiotic stress tolerance. Expression patterns of RabGAP genes in *S. lycopersicum* (sensitive species) and *S. pimpinellifolium* or *S. habrochaites* (tolerant species) in saline, drought and heat stress are represented. Higher or lower gene expressions versus the control, without stress, are shown in red and dark blue, respectively. Saline stress: *S. lycopersicum* versus *S. pimpinellifolium* (GEO accession GSE16401). RNA extraction from leaves of 6-week-old plants treated with NaCl 200 mM for 5 h [46]. Stress by drought: *S. lycopersicum* versus *S. habrochaites* (GEO accession GSE22304). Extraction of RNA from leaves of four-week-old plants, previously without irrigation for 14 days. Heat stress: *S. lycopersicum* versus *S. habrochaites* (GEO accession GSE22304). RNA extraction from leaves of four-week-old plants treated at 40 °C [47]. All values are detailed in Table S7.

To obtain more information about the role of RabGAP genes under abiotic stress conditions, we analyzed their expression profiles in response to heat, drought and salt stress in *S. lycopersicum* and compared them with those results obtained in wild relatives. In this case, the only data available for the RabGAP3, -4, -18, -20, -21a, and -21b were found in different databases (see Figure 7). During heat stress, only RabGAP4 and -20 were as strongly induced in the tolerant species as RabGAP4, -21a, and -21b were in the *S. lycopersicum*. Interestingly, under conditions of drought stress, unlike heat stress, RabGAP3, -4, -18, -20, and -21b of tolerant species versus RabGAP21a and -21b of *S. lycopersicum*, were up-regulated. Finally, under salt stress, similar expression profiles were observed in the sensitive and tolerant species. In this case, RabGAP21a and -21b were the most up-regulated genes in both species.

**Figure 8:**
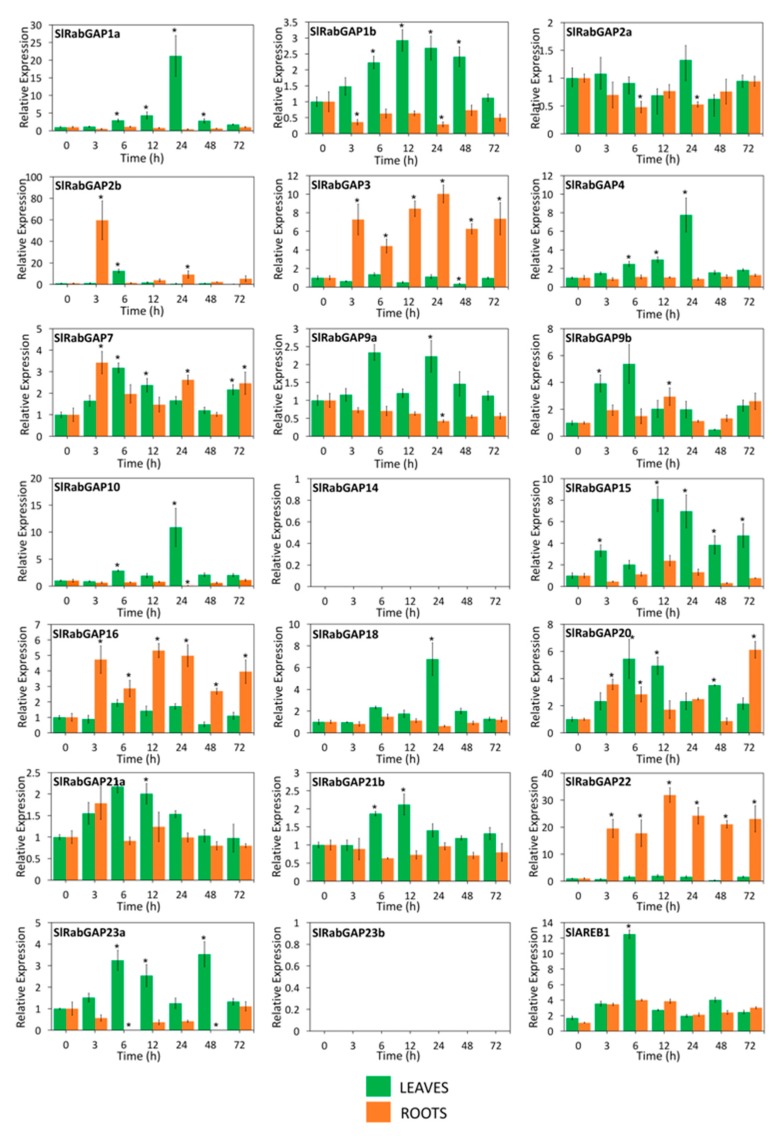
Spatio-temporal expression patterns of RabGAP genes of *S. lycopersicum* exposed to salt stress. Relative levels of SlRabGAP gene transcripts in roots (orange bar) and leaves (green bar) of 10–12-week-old *S. lycopersicum* plants were determined at 0, 3, 6, 12, 24, 48, and 72 h after stress initiation with 300 mM NaCl. The relative levels of the salt-stress marker gene (*SlAREB1*) were measured. In SlRabGAP14 and -23b, transcripts were not detected. Columns and error bars represent the mean and standard deviation for three biological and three technical replicates. * *p*-value < 0.05.

**Figure 9:**
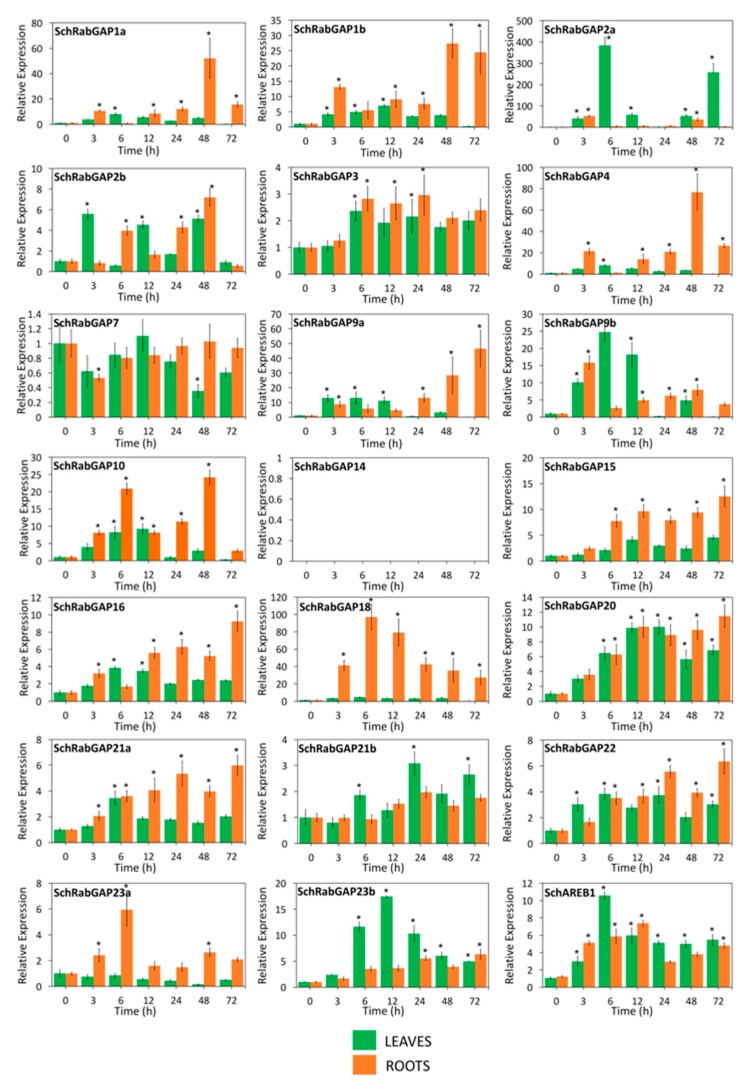
Spatio-temporal expression patterns of RabGAP genes of S. chilense exposed to salt stress. Relative levels of RabGAP gene transcripts in roots (orange bar) and leaves (green bar) of 10–12 weeks old *S. chilense* plants were determined at 0, 3, 6, 12, 24, 48, and 72 h after stress initiation with 300 mM NaCl. The relative levels of the salt-stress marker gene (SchAREB1) were measured. In SlRabGAP14, transcripts were not detected. Columns and error bars represent the mean and standard deviation for three biological and three technical replicates. * *p*-value < 0.05.

Considering the low number of genes previously analyzed, and to learn more about the stress response and determine the possible biological effect of RabGAP genes of *S. lycopersicum* during salt stress, 20 RabGAP genes representing at least one gene of each subclass were selected for expression analysis by qRT-PCR (see Figure 8). Along with the above, we used *S. chilense* as the wild and halophyte species to contrast the analyses, as shown in Figure 9. The results showed that, in both leaves and roots of *S. lycopersicum*, four genes were up-regulated (SlRabGAP2b, -7, -9b, and -20), while 10 genes were induced in leaves, and three only in roots. At early times of salt stress, RabGAP2b and -22 in roots and RabGAP9b and -20 in leaves were the most up-regulated. Similarly, at late times, RabGAP3 and -22 in roots and SlRabGAP1a, -4, -10, and -18 in leaves had the highest expression levels. Interestingly, RabGAP3, -16, and -22 in roots and RabGAP1b in leaves managed to maintain a strong and stable induction throughout the experiment. In this context, it should be also noted that pairs of paralogous genes (RabGAP1a, -1b, -2a, -2b, -9a, -9b, -21a, and -21b) showed different expression patterns.

The expression patterns of RabGAP genes in *S. chilense* revealed differences with those of *S. lycopersicum*, as seen as in Figure 9. In this species, 14 RabGAP genes significantly increased their relative expression in both leaves and roots during stress, whereas RabGAP15, -18, and -23a did it only in roots, and RabGAP21b only in leaves. We want to highlight the high and stable induction levels of RabGAP18 in roots of *S. chilense* throughout the salt stress, the inductions of RabGAP1a, -4, and -9a in roots at late times, and the large fluctuations in the expression of RabGAP2a in leaves. In addition, similar to *S. lycopersicum*, the expression levels of paralogous gene pairs also differed from the tissue in which they were expressed and their levels of induction.

## 4. Discussion

Within the Solanaceae family, the commercial tomato, *S. lycopersicum*, is the main cultivated vegetable in the world, but its sensitivity to abiotic stress negatively affects its productivity. On the other hand, wild species related to it such as *S. pimpinellifolium*, *S. habrochaites*, *S. pennellii*, and *S. chilense* are able to tolerate different degrees of abiotic stresses. A strategy of tolerance that *S. chilense* particularly uses could be associated with vesicular trafficking processes such as endocytosis, vacuolar compartmentalization of toxic ions, and damaged element recycling [21]. In this context, we have identified and characterized for the first time the family of RabGAP genes of different Solanaceaes through analyzing of the whole genome, mainly through transcriptional study. This allowed us to indicate the possible relationships between them and the components that direct the vesicular trafficking.

### 4.1. Overview of RabGAP Gene Family in the Tomato Genome

The RabGAP gene family encodes proteins capable of accelerating the hydrolysis of GTP to GDP bound to RabGTPase proteins, allowing them to direct a new round of vesicular trafficking [53]. In this work, 24 *RabGAP* genes were identified in each tomato species (*S. lycopersicum*, *S. pennellii*, and *S. pimpinellifolium*), a similar number of genes to that of other species such as rice or *A. thaliana* [15]. However, according to motifs and multiple alignment analyses, the results suggest that four proteins, including RabGAP23b, should not have RabGAP activity due to the absence of a conserved Arginine residue. In this regard, it has been evidenced that a point mutation on this residue in OsGAP1 (R450A) causes a severe loss of this activity against two RabGTPases—OsRab11 and OsRab8a—revealing its importance in catalytic activity [24].

Interestingly, the subclasses I and II of the RabGAP family are composed of six and two RabGAP genes, respectively. In addition, both families are potentially exclusive to plants and it is predicted that their evolution is an adaptation mechanism due to the loss of function related to the RasGAP family [15]. From the above, possible coevolution mechanisms between RabGTPases and RabGAPs, which have sculpted the endomembrane system, have been suggested [54]. On the other hand, the equivalent number of members of the RabGAP family between tomato and evolutionarily distant species, such as *A. thaliana* or rice, is an important question to raise. In this sense, it has been suggested that duplication and diversification events from five ancestral RabGAP genes have been the mechanisms that have kept the number of genes constant [15,28,55]. The exon-intron structure also provides relevant information to understand the evolution process of the RabGAP family. In this analysis, we found paralogous genes from subclasses I (RabGAP23b and -15), III (RabGAP21b and -21b), and VIII (RabGAP9a and -9b) located on chromosomes 7 and 12. The arrangements and size of exons and introns of these genes supports the hypothesis of an important segmental duplication event between both chromosomes, which has also been reported for members of the B-BOX, LEA, and GRAS families of *S. lycopersicum* [56,57,58]. Together, these data suggest that events of segmental duplications have played an important role in the gene evolution of the RabGAP gene family.

### 4.2. The New Subfamily VI and Rab3GAP Could Play an Important Role in Autophagy and Stress Tolerance

Regarding the characteristics of other identified genes, we found two new members of subclass VI with a Rab3GAP domain whose functional roles in plants have not yet been determined, see in Figure 1 and Table 1. However, homolog genes in animal cells participate in: (i) the deactivation of Rab3 [14], a homolog of RabD in plants, (ii) interaction with ATG8 in autophagy processes [59,60], (iii) or activation of a RabGTPase of subfamily C, acting as a guanine exchange factor in degradative pathways and macro-autophagy [61]. Intriguingly, a RabD of plants (RABD2A) localizes with proteins associated with salt-stress tolerance capacity, such as EHD1 or SYP41 [62,63]. In a similar way to expression patterns of Rab3GAP1, Rab3GAP2, and RabGAP10 of *S. lycopersicum*, the autophagy is a mechanism rapidly induced by salt stress [64]. These interesting characteristics suggest that the proteins of subclass VI can probably interact with RabD or ATG8 and play an important role during salt stress in tomato.

### 4.3. Endocytosis-Associated RabGAPs Could Be Mediating the Salt Stress Tolerance in Tomato

In general, the different RabGAP proteins of *S. lycopersicum* identified in this work were located in the nucleus or cytoplasm. Particularly, the predicted nuclear localization was consistent among RabGAP22 from *S. lycopersicum* and *A. thaliana* [65]. The patterns of expression of both genes are also similar. In both species, RabGAP22 is expressed in various tissues and stages of development, such as flowers, roots, or vegetative meristems. Along with this, RabGAP22 from *A. thaliana* can interact with peroxisome proteins such as AGT1 and be associated with photorespiration processes or adaptation mechanisms against abiotic stress [66]. According to this, the homolog of AtRabGAP22 in *S. lycopersicum* could have an important role in tomato roots when they are subjected to salt stress. On the other hand, the predicted subcellular location of RabGAP21a from *S. lycopersicum*, with respect to its rice homolog, OsGAP1 [23], is also coherent. In this case, the data suggest that SlRabGAP21a could be localized in the Trans-Golgi network and the pre-vacuolar compartments. Equally, it is interesting to observe that the expression patterns of OsGAP1 and RabGAP21a of *S. lycopersicum* are similar in leaves and roots when plants are subjected to salt stress [24]. These data suggest that RabGAP21 could also have an important role in a trafficking pathway that mobilizes transporters or proteins associated with the vacuole. Additionally, SlRabGAP21a could potentially interact with the TVP38 protein, which has been associated with the stability of the membranes [67]. The results for RabGAP18 are also consistent with the fact that the endocytosis mechanism is an inducible process against salt stress and regulated for RabGTPases of the subfamily-F [20,22]. To date, the RabGAP proteins that regulate this subfamily-F are unknown, however, the protein-protein interaction analysis suggested that RabGAP18 could be an exclusive regulator of this family. Interestingly, RabGAP18 is also strongly induced in the roots of *S. chilense* when they are exposed to salt stress in a similar manner to SchRabGDI1, whose overexpression in *A. thaliana* can increase its tolerance to salt stress [21]. In general, the various antecedents support the idea that RabGAP have a certain degree of affinity for RabGTPases, but the results equally show a high degree of promiscuity for some of them. In this sense, further analyses and experiments are needed to establish the vesicular trafficking routes in which the members of the RabGAP family of *S. lycopersicum* participate in order to finally understand their physiological functions during the abiotic stress.

## 5. Conclusions

In conclusion, we have identified 24 RabGAP genes in each *S. lycopersicum*, *S. pennellii*, and *S. pimpinellifolium* and classified them into seven subclasses. Two proteins with Rab3GAP domain were identified for the first time. All of them have the TBC domain characteristic of the RabGAP family. Evolutionarily segmental duplication and purifying selection have maintained constant the number and function of these genes in the *S. lycopersicum* genome. The results presented here show that the RabGAP proteins, such as RabGAP18 or RabGAP22, could be associating to different intracellular vesicular trafficking pathways and that the genes that encode them can change their expression depending on both endogenous and environmental stimuli. In this sense, the study of the regulation mechanisms of gene expressions, as well as the specific interactions with RabGTPase proteins could provide important information to understand their participation in tolerating abiotic stress in tomato species.

## Figures and Tables

**Table 1:** List of RabGTPase activating proteins (RabGAP) genes found in domesticated and wild tomatoes and their physical and chemical characteristics. Data obtained from Sol Genomics Network database. Gene prefixes represent *Solanum lycopersicum* (Sl), *Solanum pennellii* (Spe), and *Solanum pimpinellifolium* (Spi). Subcellular location: ML2, MultiLoc2; WPS, Wolf PSORT. Nuc, Nuclear; Cyt, Cytoplasmic; Chl, Chloroplast; MP, Plasma Membrane; ER, Endoplasmic Reticulum; and Mit, Mitochondria.

Gene Name	Gene ID	Chr	Chromosome Location		Length (aa)	Mol. Wt. (kDa)	Isolelec. Point	Conserved Domain	Domain Location		GRAVY	Subcellular Location		
			Start	End					Start	End		CELLO	ML2	WPS
*SlRab3GAP1*	Solyc07g064740	7	66763414	66771800	673	74.853	5.14	Rab3-GTPase_cat	377	530	−0.478	Nuc	Cyt	Nuc
*SlRab3GAP2*	Solyc06g082200	6	48103293	48118442	942	106.62	4.93	Rab3-GTPase_cat	555	720	−0.386	Cyt	Cyt	Nuc
*SlRabGAP1a*	Solyc05g053710	5	63747738	63754496	393	43.78	5.38	TBC	144	393	−0.486	Nuc	Cyt	Nuc
*SlRabGAP1b*	Solyc09g092070	9	71223957	71231836	418	47.161	5.98	TBC	88	387	−0.283	PM	Cyt	Nuc
*SlRabGAP2a*	Solyc07g008840	7	3803764	3808890	822	90.686	4.98	TBC	16	403	−0.491	Nuc	Cyt	Nuc
*SlRabGAP2b*	Solyc02g014450	2	16619223	16626117	726	80.876	5.31	TBC	3	277	−0.544	Nuc	Cyt	Chl
*SlRabGAP3*	Solyc06g008250	6	2120441	2130757	432	49.448	8.53	TBC	71	348	−0.352	Cyt	Cyt	Cyt
*SlRabGAP4*	Solyc11g005010	11	15822	26251	447	51.433	5.53	TBC	147	403	−0.364	Nuc	Cyt	Nuc
*SlRabGAP5*	Solyc09g007160	9	779711	793133	797	90.48	5.08	TBC	220	457	−0.625	Cyt	Cyt	Cyt
*SlRabGAP6*	Solyc04g009560	4	2961142	2970580	828	93.555	4.79	TBC	244	482	−0.581	Cyt	Cyt	Chl
*SlRabGAP7*	Solyc03g058470	3	24712188	24716135	571	64.49	5.58	TBC	182	427	−0.46	Nuc	Cyt	Chl
*SlRabGAP9a*	Solyc07g049580	7	59919122	59923736	394	44.936	8.38	TBC	108	325	−0.154	Cyt	Cyt	Nuc
*SlRabGAP9b*	Solyc12g005930	12	554207	559357	394	44.931	7.21	TBC	108	325	−0.163	Cyt	Cyt	Cyt
*SlRabGAP10*	Solyc07g062450	7	65184937	65189399	455	50.645	8.59	Rab3GAP2_N	28	416	−0.07	PM	Cyt	Chl
*SlRabGAP14*	Solyc02g071420	2	40880409	40882735	459	53.267	5.93	TBC	122	421	−0.376	Nuc	Cyt	Cyt
*SlRabGAP15*	Solyc12g005220	12	141022	146306	438	50.259	7.99	TBC	61	344	−0.387	Cyt	Nuc	Nuc
*SlRabGAP16*	Solyc09g066420	9	64831553	64843975	485	54.757	5.61	TBC	78	392	−0.514	Nuc	Cyt	Nuc
*SlRabGAP18*	Solyc01g101090	1	90939383	90945694	352	40.937	8.97	TBC	80	297	−0.169	Cyt	Cyt	Cyt
*SlRabGAP20*	Solyc06g053190	6	35918577	35924309	553	62.418	7.03	TBC	108	497	−0.457	Nuc	Nuc	Chl
*SlRabGAP21a*	Solyc12g009610	12	2864130	2875362	656	75.17	5.32	TBC	351	589	−0.49	Nuc	Cyt	Nuc
*SlRabGAP21b*	Solyc07g008000	7	2697439	2709902	678	78.042	5.78	TBC	374	612	−0.518	Nuc	Cyt	Nuc
*SlRabGAP22*	Solyc03g082590	3	52516500	52523765	559	62.937	5.84	TBC	115	495	−0.425	Nuc	ER	Chl
*SlRabGAP23a*	Solyc10g006440	10	1046336	1054965	450	51.287	6.09	TBC	68	359	−0.483	Cyt	Cyt	Nuc
*SlRabGAP23b*	Solyc07g064230	7	66456796	66462000	425	48.5	8.57	TBC	60	333	−0.409	Cyt	Cyt	Cyt
*SpeRab3GAP1*	Sopen07g032940	7	77919830	77932192	674	74.805	5.04	Rab3-GTPase_cat	359	514	−0.48	Nuc	Cyt	Nuc
*SpeRab3GAP2*	Sopen06g033580	6	59015664	59042332	942	106.478	4.85	Rab3-GTPase_cat	555	720	0.394	Cyt	Cyt	Nuc
*SpeRabGAP1a*	Sopen05g032190	5	75703069	75712722	483	53.886	5.56	TBC	252	442	−0.367	Nuc	Cyt	Nuc
*SpeRabGAP1b*	Sopen09g034940	9	82925996	82937126	493	55.01	5.76	TBC	269	459	−0.357	Nuc	Cyt	Nuc
*SpeRabGAP2a*	Sopen07g004770	7	4263306	4270907	822	90.576	4.91	TBC	106	355	−0.504	Nuc	Cyt	Nuc
*SpeRabGAP2b*	Sopen02g003110	2	7156270	7165642	763	84.747	5.52	TBC	15	299	−0.5	Nuc	Cyt	Chl
*SpeRabGAP3*	Sopen06g003110	6	2485987	2500959	432	49.464	8.34	TBC	177	323	−0.367	Cyt	Cyt	Cyt
*SpeRabGAP4*	Sopen11g001030	11	25457	41033	447	51.461	5.34	TBC	147	400	−0.372	Nuc	Cyt	Nuc
*SpeRabGAP5*	Sopen09g002030	9	807911	827341	798	90.531	4.98	TBC	289	449	−0.613	Cyt	Cyt	Cyt
*SpeRabGAP6*	Sopen04g004720	4	3140714	3154812	829	93.647	4.71	TBC	245	480	−0.577	Cyt	Cyt	Chl
*SpeRabGAP7*	Sopen03g012120	3	18722571	18792333	692	78.416	6.14	TBC	375	525	−0.455	Nuc	Cyt	Nuc
*SpeRabGAP9a*	Sopen07g024980	7	70500266	70507267	395	44.936	7.42	TBC	108	322	−0.154	Cyt	Cyt	Nuc
*SpeRabGAP9b*	Sopen12g001880	12	657246	665470	395	44.947	6.75	TBC	108	322	−0.17	Cyt	Cyt	Cyt
*SpeRabGAP10*	Sopen07g030690	7	76219201	76225527	456	50.618	7.34	Rab3GAP2_N	28	416	−0.063	PM	Cyt	Chl
*SpeRabGAP14*	Sopen02g020470	2	44244421	44247921	414	48.169	5.36	TBC	123	403	−0.454	Nuc	Cyt	Cyt
*SpeRabGAP15*	Sopen12g001200	12	187739	195817	438	50.174	7.08	TBC	174	333	−0.36	Cyt	Nuc	Nuc
*SpeRabGAP16*	Sopen09g028140	9	76268366	76277038	468	53.047	5.22	TBC	203	362	−0.499	Nuc	Cyt	Nuc
*SpeRabGAP18*	Sopen01g044540	1	101408643	101416953	352	40.937	8.28	TBC	80	294	−0.169	Cyt	Cyt	Cyt
*SpeRabGAP20*	Sopen06g018250	6	45454478	45463520	553	62.472	6.91	TBC	354	470	−0.453	Nuc	Chl	Chl
*SpeRabGAP21a*	Sopen12g004600	12	3139864	3156889	656	75.112	5.22	TBC	351	586	−0.48	Nuc	Cyt	Nuc
*SpeRabGAP21b*	Sopen07g003880	7	2957429	2975101	678	78.098	5.69	TBC	374	609	−0.53	Nuc	Cyt	Nuc
*SpeRabGAP22*	Sopen03g022350	3	53888278	53898904	540	61.034	5.93	TBC	337	455	−0.454	Nuc	Mit	Chl
*SpeRabGAP23a*	Sopen10g002390	10	1110898	1124727	442	50.427	6.09	TBC	60	322	−0.398	Mit	Cyt	Cyt
*SpeRabGAP23b*	Sopen07g032380	7	77586099	77593750	439	50.223	8.65	TBC	60	322	−0.398	Mit	Cyt	Cyt
*SpiRab3GAP1*	Sopim07g064740	7	−	−	673	74.853	5.14	Rab3-GTPase_cat	359	514	−0.478	Nuc	Cyt	Nuc
*SpiRab3GAP2*	Sopim06g082200	6	−	−	907	102.787	4.83	Rab3-GTPase_cat	555	720	−0.391	Cyt	Cyt	Nuc
*SpiRabGAP1a*	Sopim05g053710	5	−	−	393	43.78	5.38	TBC	145	380	−0.487	Nuc	Cyt	Nuc
*SpiRabGAP1b*	Sopim09g092070	9	−	−	418	47.161	5.98	TBC	194	384	−0.283	PM	Cyt	Cyt
*SpiRabGAP2a*	Sopim07g008840	7	−	−	822	90.686	4.98	TBC	106	355	−0.491	Nuc	Cyt	Nuc
*SpiRabGAP2b*	Sopim02g014450	2	−	−	726	80.876	5.31	TBC	15	252	−0.544	Nuc	Cyt	Chl
*SpiRabGAP3*	Sopim06g008250	6	−	−	432	49.448	8.53	TBC	177	323	−0.352	Cyt	Cyt	Cyt
*SpiRabGAP4*	Sopim11g005010	11	−	−	447	51.432	5.4	TBC	147	400	−0.364	Nuc	Cyt	Nuc
*SpiRabGAP5*	Sopim09g007160	9	−	−	797	90.48	5.08	TBC	289	449	−0.625	Cyt	Cyt	Cyt
*SpiRabGAP6*	Sopim04g009560	4	−	−	828	93.555	4.79	TBC	244	479	−0.581	Cyt	Cyt	Chl
*SpiRabGAP7*	Sopim03g058470	3	−	−	571	64.49	5.58	TBC	254	404	−0.46	Nuc	Cyt	Chl
*SpiRabGAP9a*	Sopim07g049580	7	−	−	394	44.936	8.38	TBC	108	322	−0.154	Cyt	Cyt	Nuc
*SpiRabGAP9b*	Sopim12g005930	12	−	−	394	44.931	7.21	TBC	108	322	−0.163	Cyt	Cyt	Cyt
*SpiRabGAP10*	Sopim07g062450	7	−	−	455	50.645	8.59	Rab3GAP2_N	28	416	−0.067	PM	Cyt	Chl
*SpiRabGAP14*	Sopim02g071420	2	−	−	459	53.267	5.93	TBC	123	410	−0.376	Nuc	Cyt	Cyt
*SpiRabGAP15*	Sopim12g005220	12	−	−	438	50.259	7.99	TBC	174	323	−0.387	Cyt	Nuc	Nuc
*SpiRabGAP16*	Sopim09g066420	9	−	−	485	54.757	5.61	TBC	220	379	−0.513	Nuc	Cyt	Nuc
*SpiRabGAP18*	Sopim01g101090	1	−	−	352	40.937	8.97	TBC	80	294	−0.169	Cyt	Cyt	Cyt
*SpiRabGAP20*	Sopim06g053190	6	−	−	553	62.418	7.03	TBC	354	470	−0.457	Cyt	Nuc	Chl
*SpiRabGAP21a*	Sopim12g009610	12	−	−	656	75.17	5.32	TBC	351	586	−0.49	Nuc	Cyt	Nuc
*SpiRabGAP21b*	Sopim07g008000	7	−	−	720	78.042	5.56	TBC	374	609	−0.518	Nuc	Cyt	Nuc
*SpiRabGAP22*	Sopim03g082590	3	−	−	559	62.937	5.84	TBC	357	475	−0.425	Nuc	ER	Chl
*SpiRabGAP23a*	Sopim10g006440	10	−	−	450	51.287	6.09	TBC	184	342	−0.483	Cyt	Cyt	Nuc
*SpiRabGAP23b*	Sopim07g064230	7	−	−	425	48.5	8.57	TBC	173	308	−0.409	Cyt	Cyt	Cyt

**Table 2:** Evolutionary parameters for duplicated RabGAP genes. Table shows Ka/Ks ratios, duplication and selection types and time of divergence.

Gene Pairs Duplicated		Duplication Type	Ka	Ks	Ka/Ks	Selection	Time (mya)
*SlRabGAP23a*	*SlRabGAP23b*	Segmental	0.1	0.52	0.192	Purify	17.3
*SlRabGAP23b*	*SlRabGAP15*	Segmental	0.12	0.55	0.218	Purify	18.3
*SlRabGAP20*	*SlRabGAP22*	Segmental	0.18	0.62	0.290	Purify	20,6
*SlRabGAP21a*	*SlRabGAP21b*	Segmental	0.1	0.71	0.140	Purify	23.6
*SlRabGAP1a*	*SlRabGAP1b*	Segmental	0.11	0.69	0.159	Purify	23
*SlRabGAP9a*	*SlRabGAP9b*	Segmental	0.07	0.49	0.142	Purify	16.3
*SlRabGAP5*	*SlRabGAP6*	Segmental	0.09	0.86	0.171	Purify	28.6
*SlRabGAP2a*	*SlRabGAP2b*	Segmental	0.11	0.46	0.230	Purify	15.3
*SlRabGAP3*	*SlRabGAP16*	Segmental	0.13	0.63	0.206	Purify	21

Ka: number of nonsynonymous substitutions per non-synonymous site; Ks: number of synonymous substitutions per synonymous site. Mya: million years ago.

## Supplementary Materials

The following are available online at https://www.mdpi.com/2073-4425/10/9/638/s1, Figure S1: Multiple alignment of RabGAP sequences. RabGAP amino-acid sequences of *S. lycopersicum* and *A. thaliana*. In green, conserved amino-acids associated with catalytic activity, File S1: Expression patterns of RabGAP genes in different tissues and development stages of tomato (Tomato eFP Browser), Table S1: Primers used in this study, Table S2: Regular expression profile of the conserved motifs defined by MEME of SlRabGAP, Table S3: ID of *RabGAP* genes in *A. thaliana* used in phylogenetic analysis, Table S4: Protein-Protein interaction networks predicted with STRING database, Table S5: Relative expression of RabGAP genes in different tissues and development stages of *S. lycopersicum* and *S. pennellii*. Log2 (ratio), Table S6: Relative expression of RabGAP genes among different tomato species (normalized counts per million), Table S7: Relative expression of RabGAP genes from contrasting tomato species in different types of abiotic stresses.
