# Supplementary material for: The RabGAP Gene Family in Tomato (Solanum lycopersicum) and Wild Relatives: Identification, Interaction Networks, and Transcriptional Analysis during Plant Development and in Response to Salt Stress
Source: Genes (Basel). 2019 Aug 23;10(9):638. doi: 10.3390/genes10090638 (PMC6770026; doi:10.3390/genes10090638)
Supplement: Supplementary file 1 [file genes-10-00638-s001.zip › Supplementary File/File S1.docx]

File S1. Comparative transcriptome of *RabGAP* genes in different tissues and development stages. Data obtained from the Toronto BAR Database.

***Rab3GAP1***

***
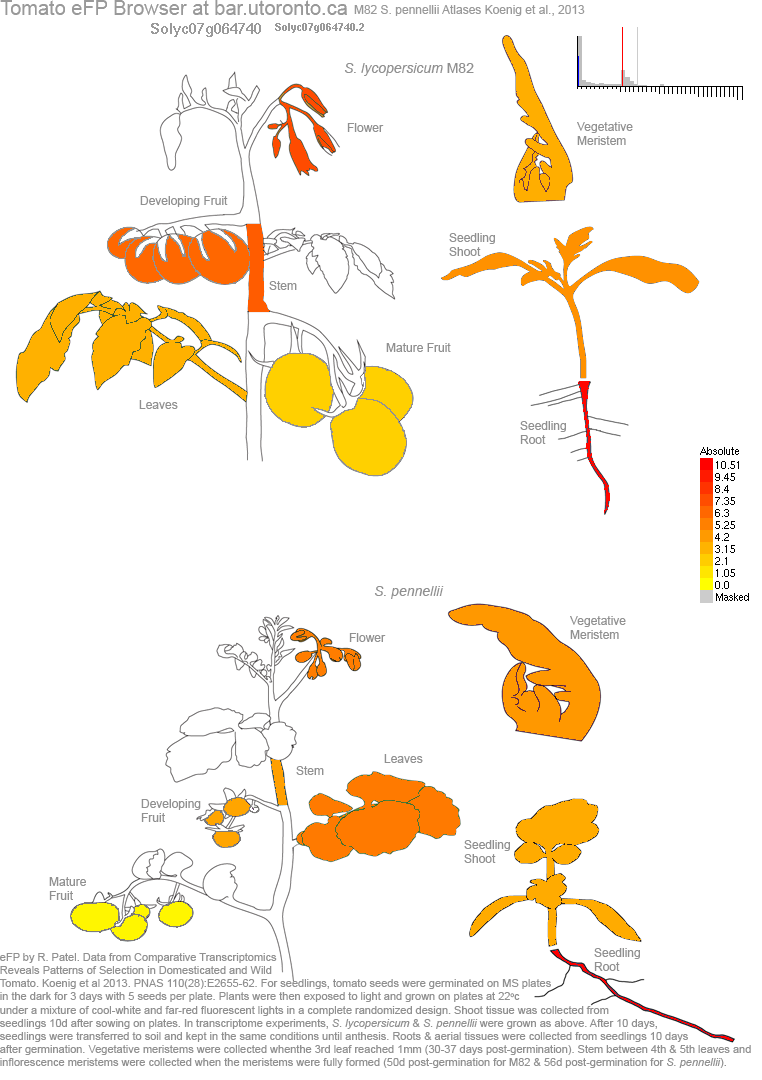
***

***Rab3GAP2***

***
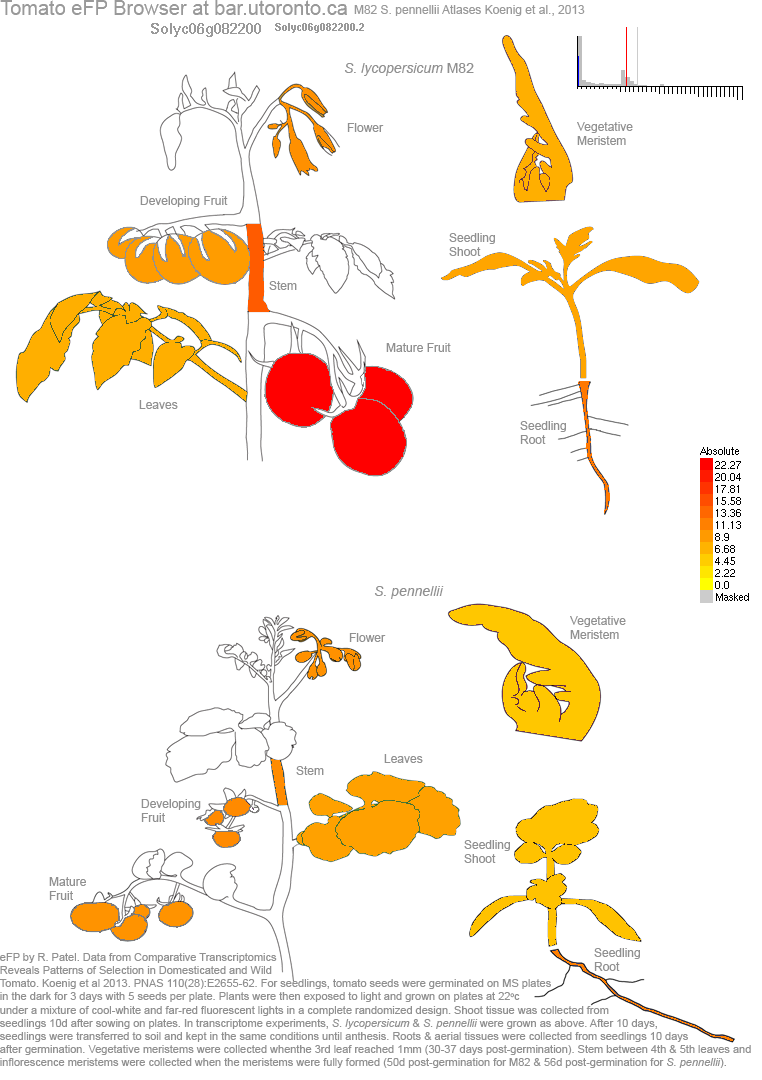
***

***RabGAP1a***

***
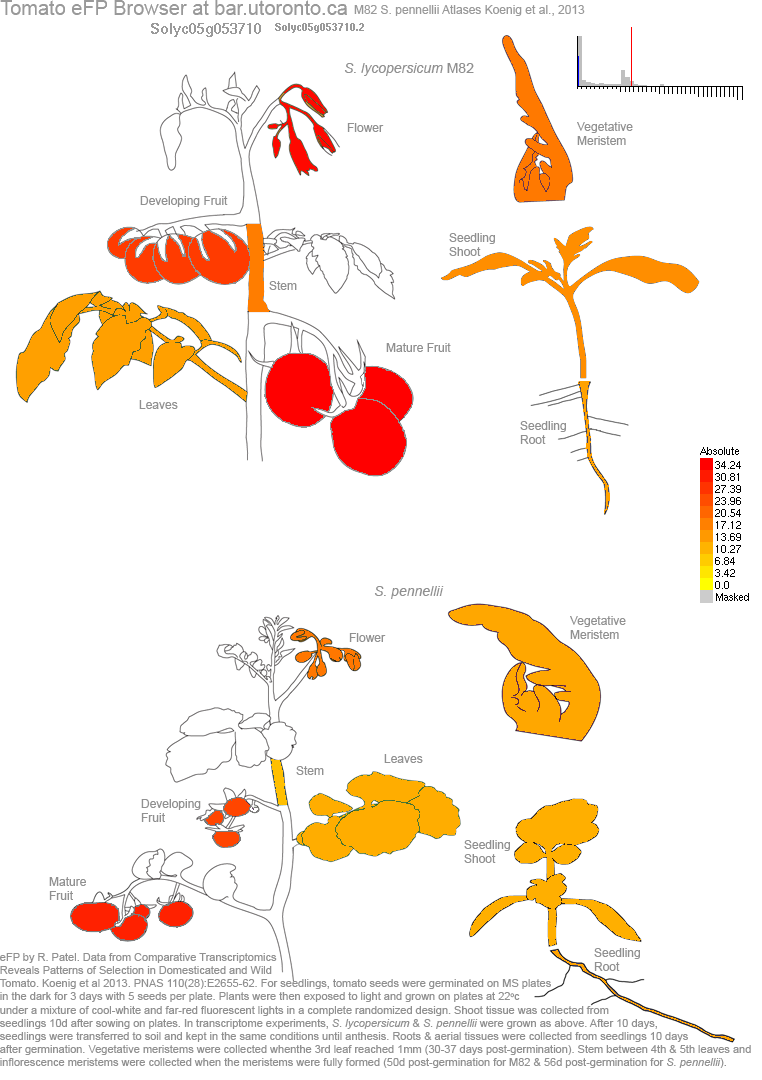
***

***RabGAP1b***

***
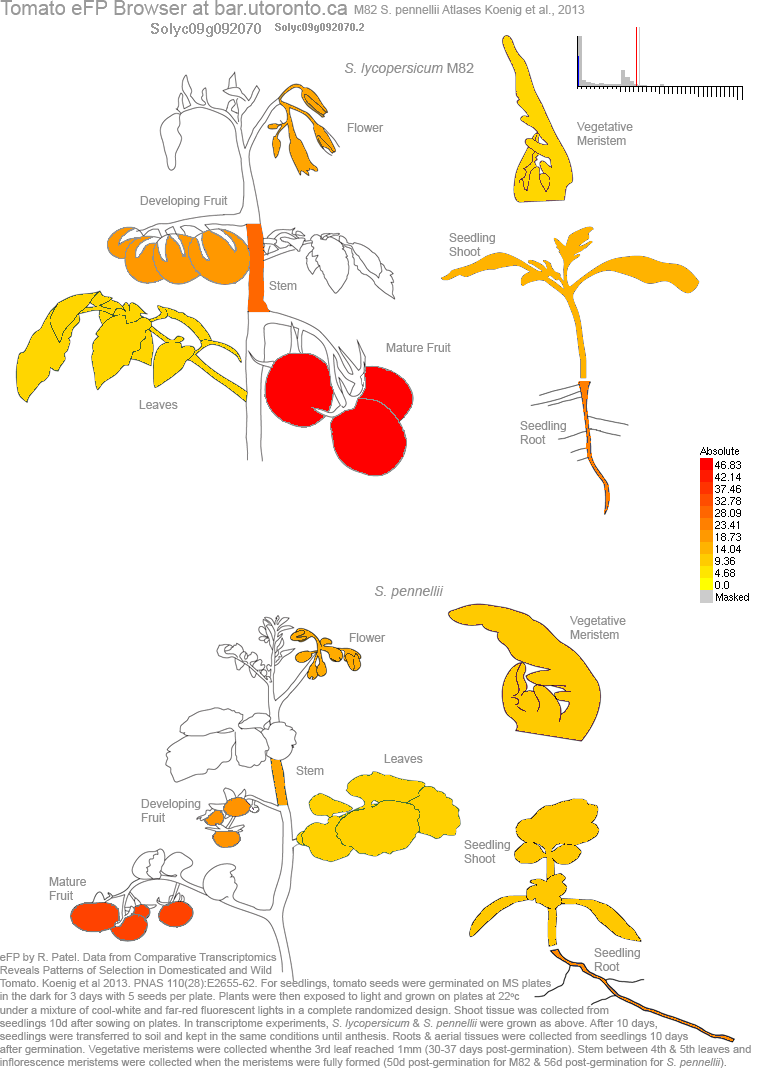
***

***RabGAP2a***

***
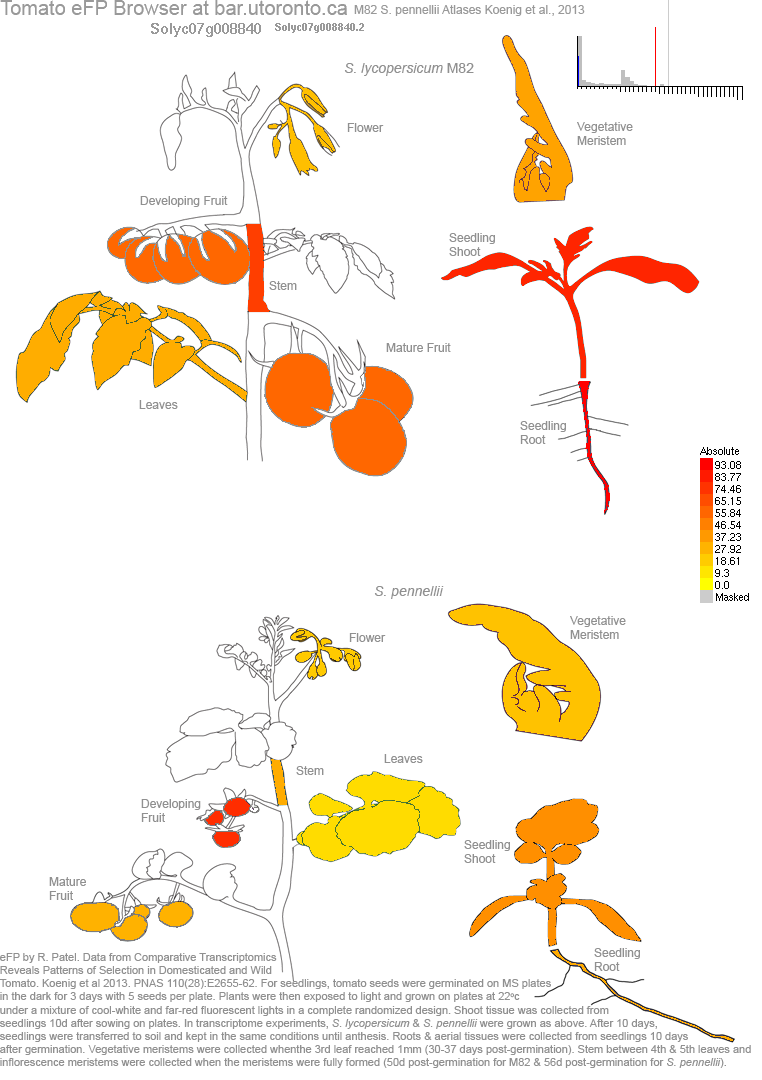
***

***RabGAP2b***

***
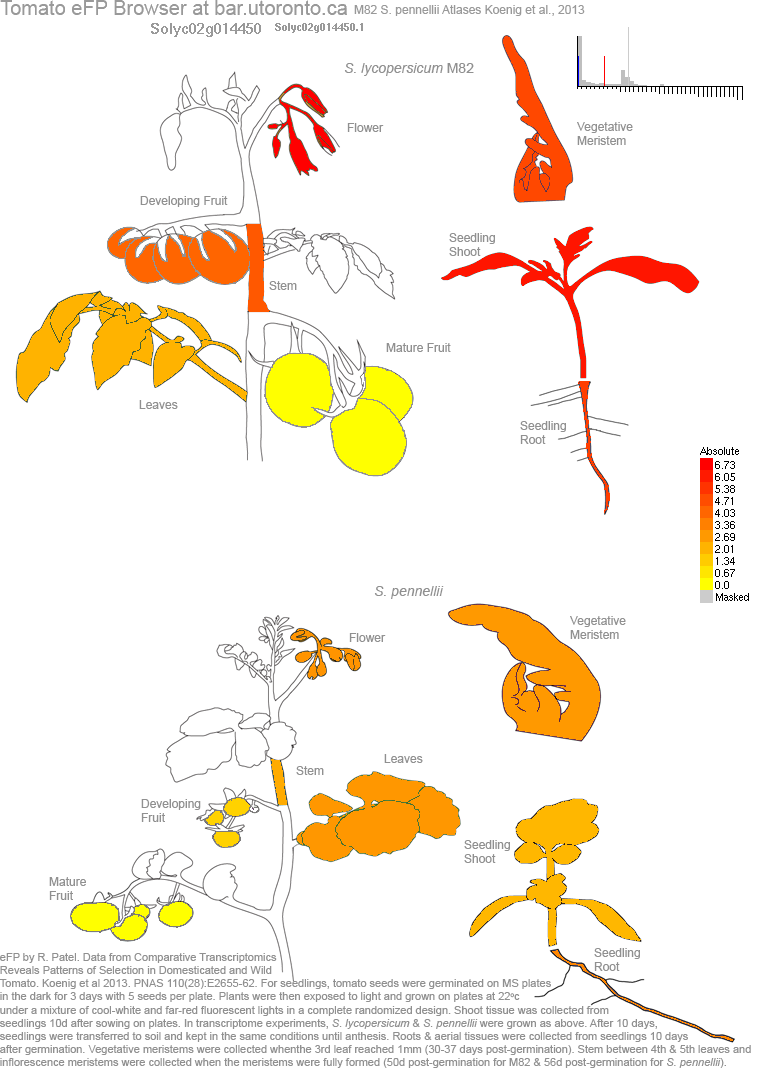
***

***RabGAP3***

***
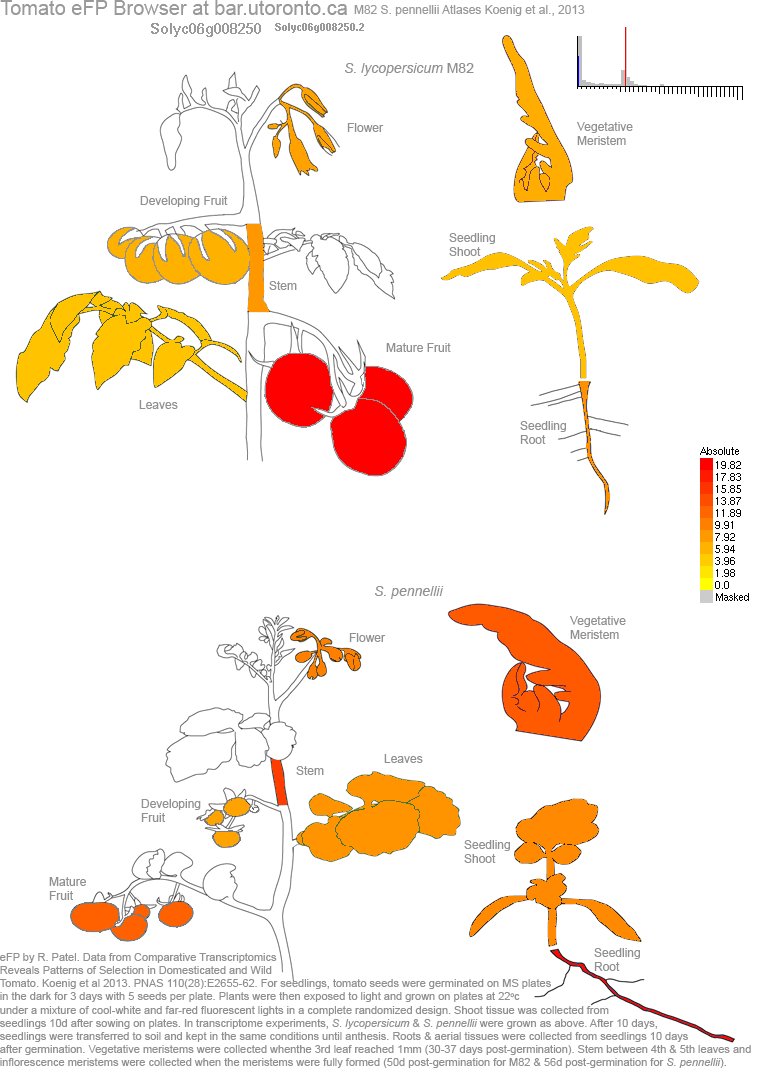
***

***RabGAP4***

***
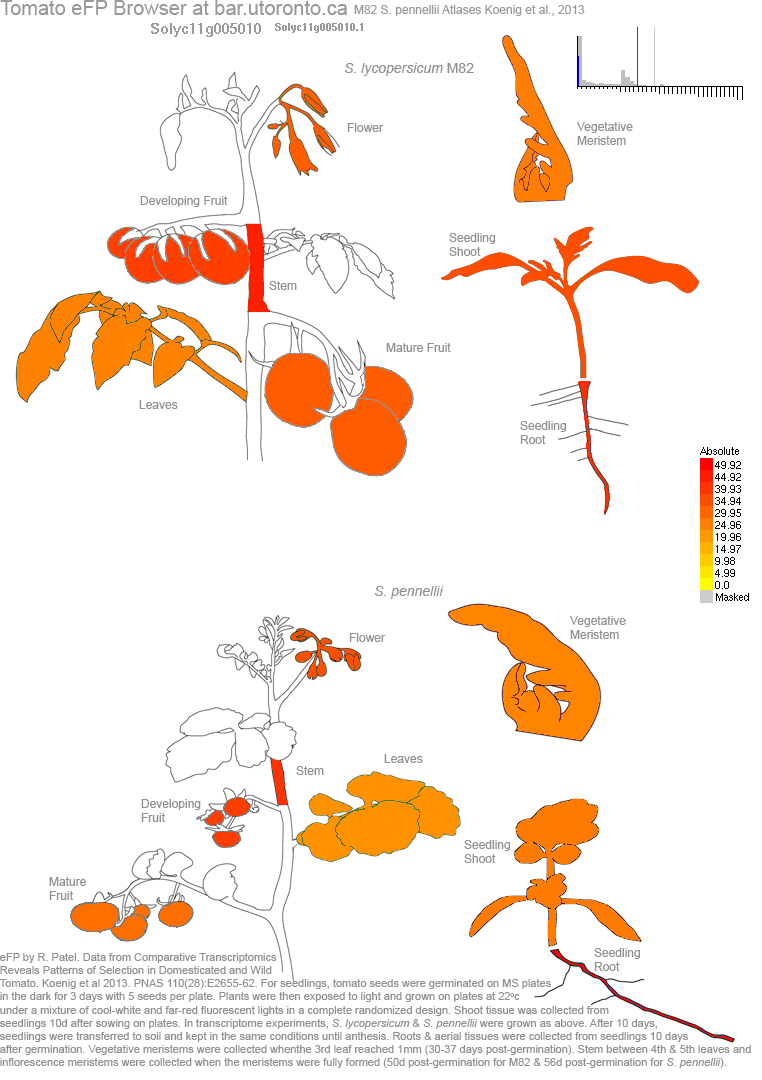
***

***RabGAP5***

***
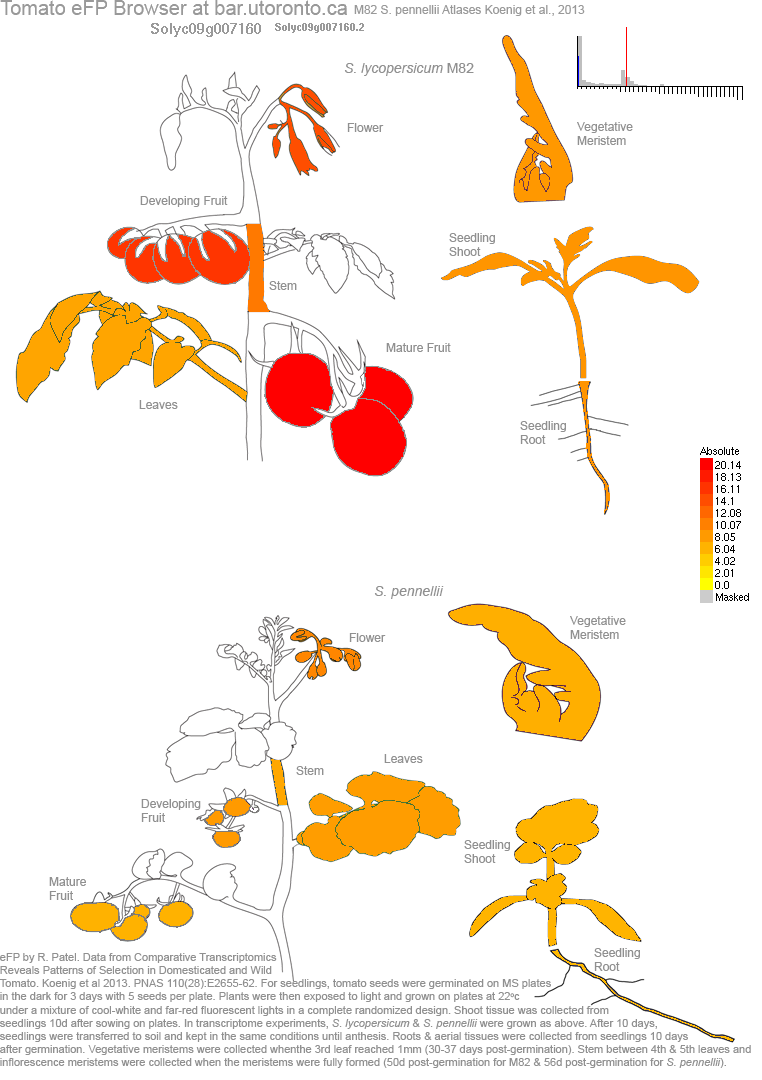
***

***RabGAP6***

***
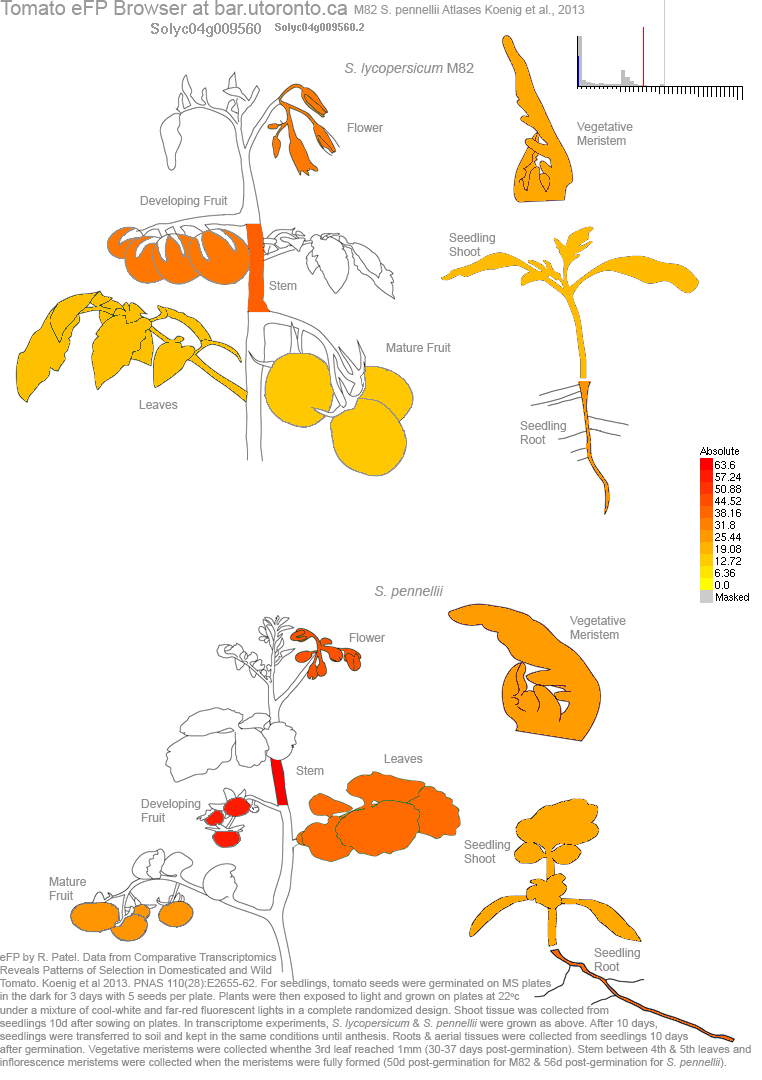
***

***RabGAP7***

***
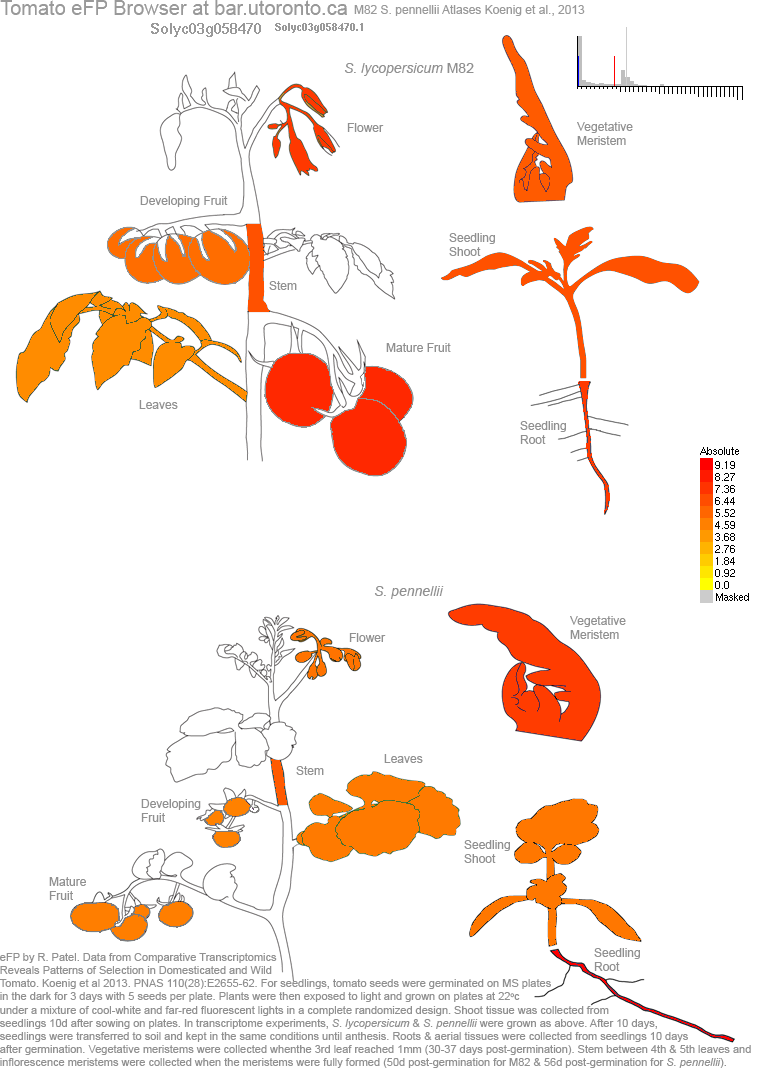
***

***RabGAP9a***

***
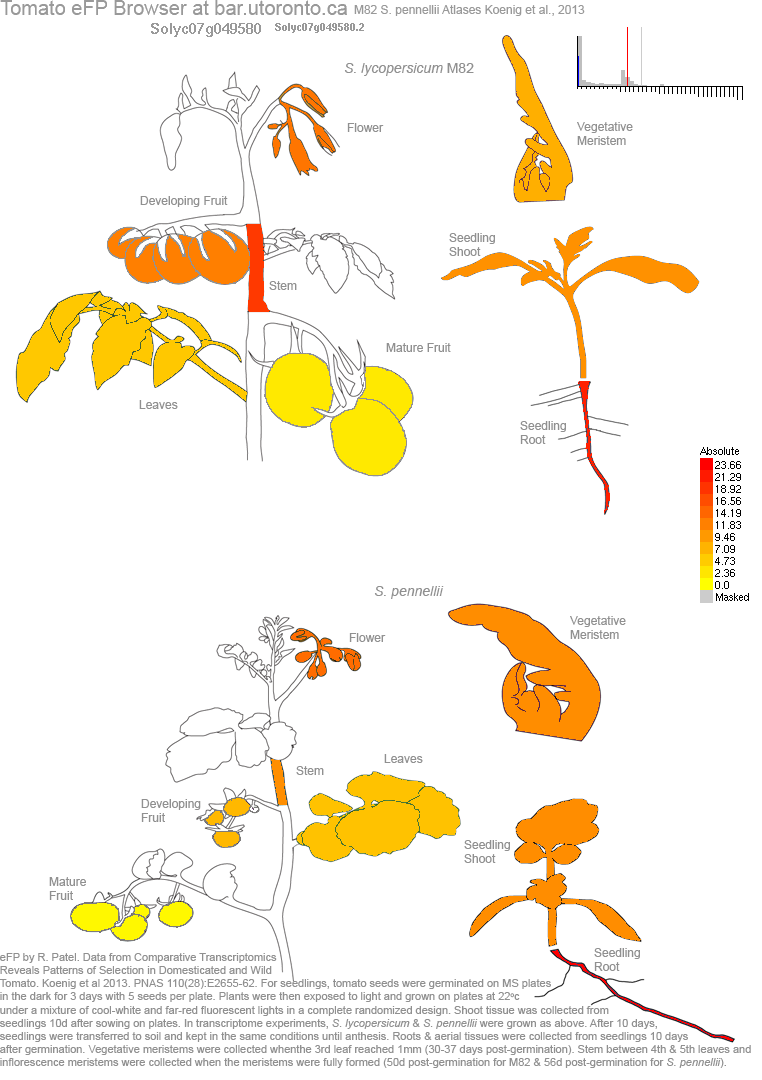
***

***RabGAP9b***

***
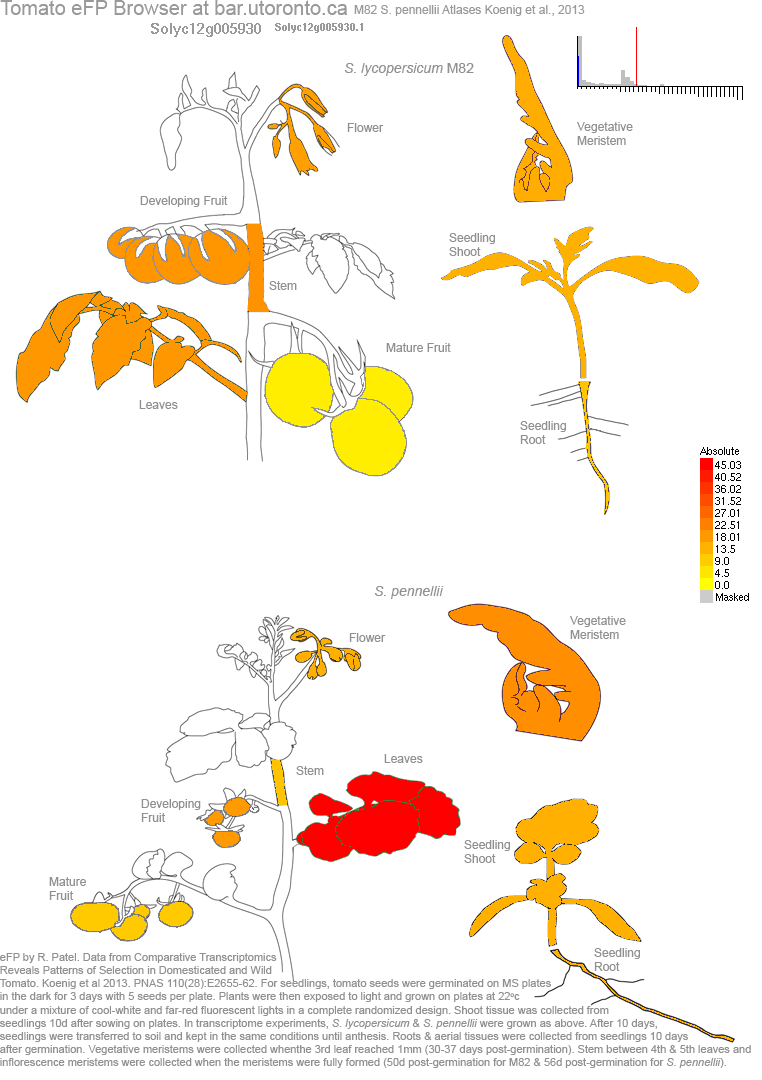
***

***RabGAP10***

***
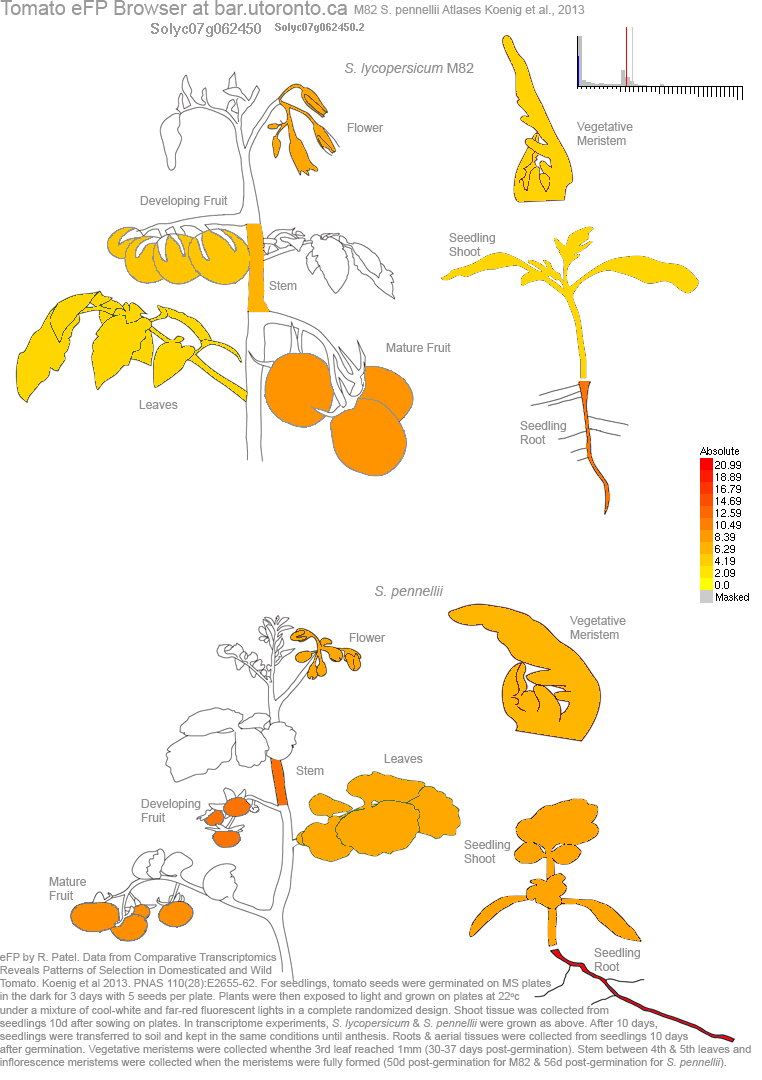
***

***RabGAP14***

***
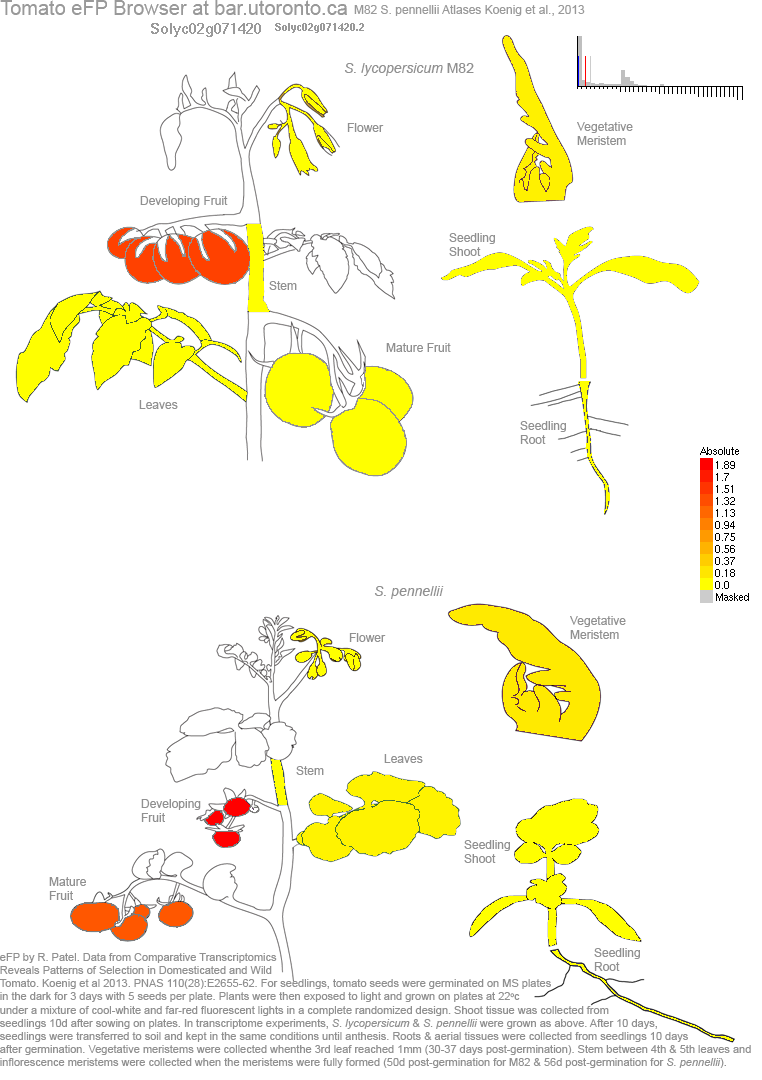
***

***RabGAP15***

***
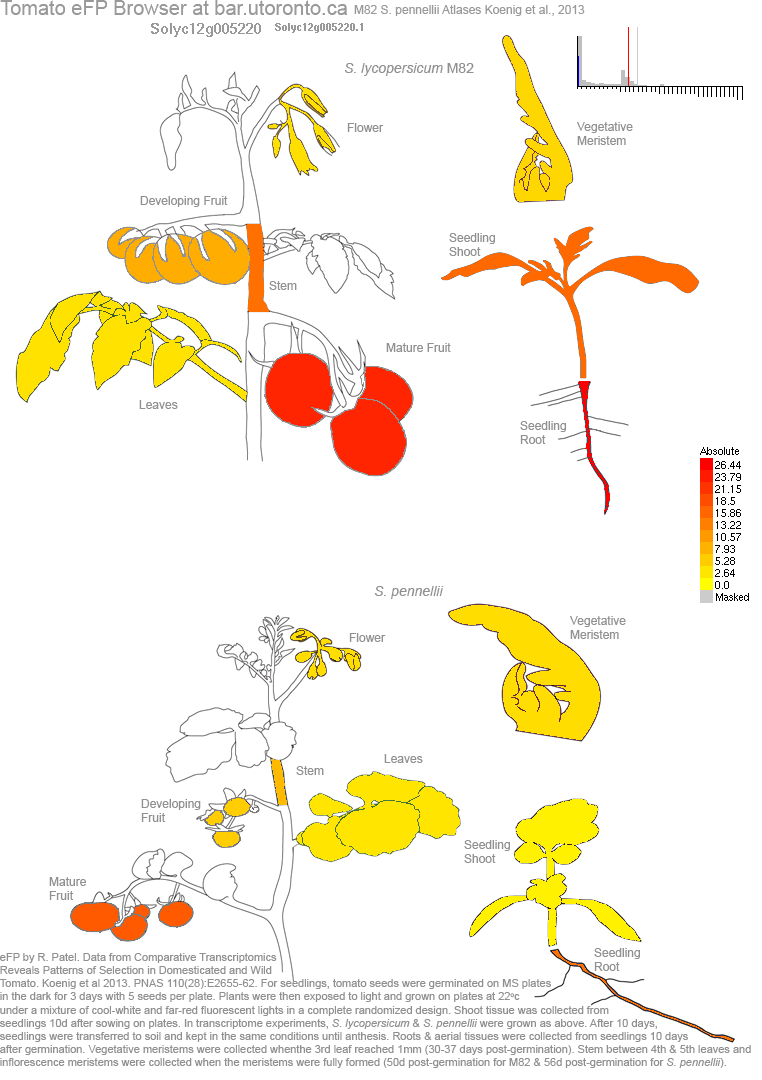
***

***RabGAP16***

***
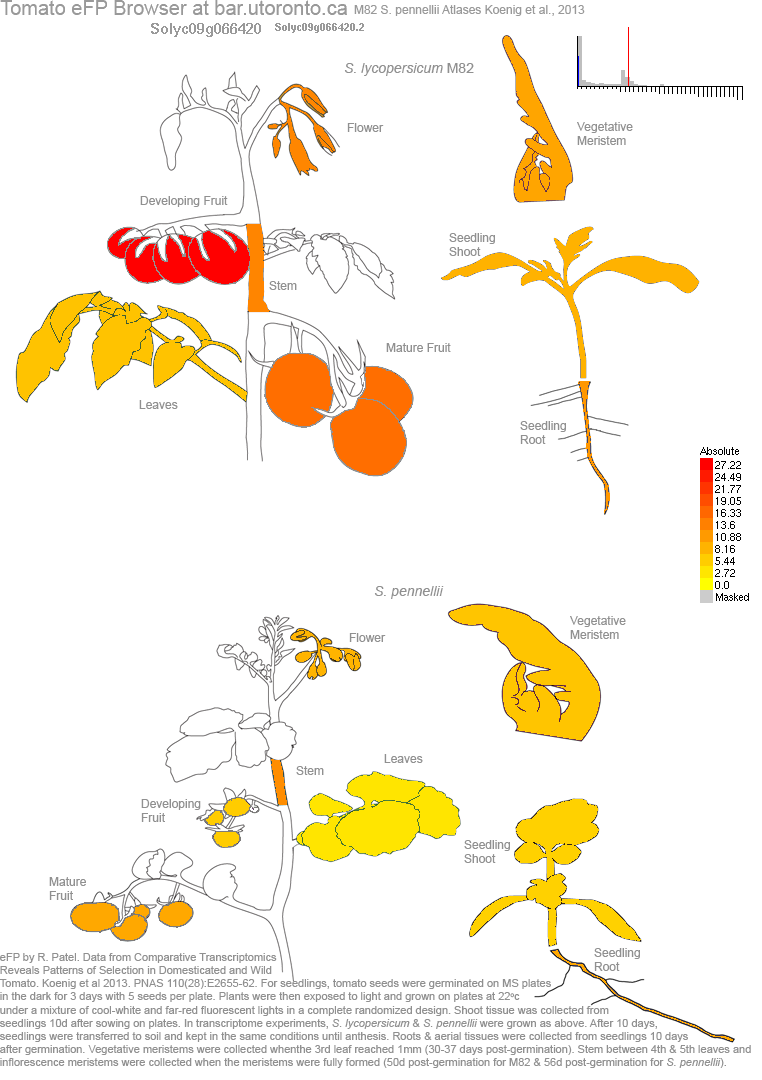
***

***RabGAP18***

***
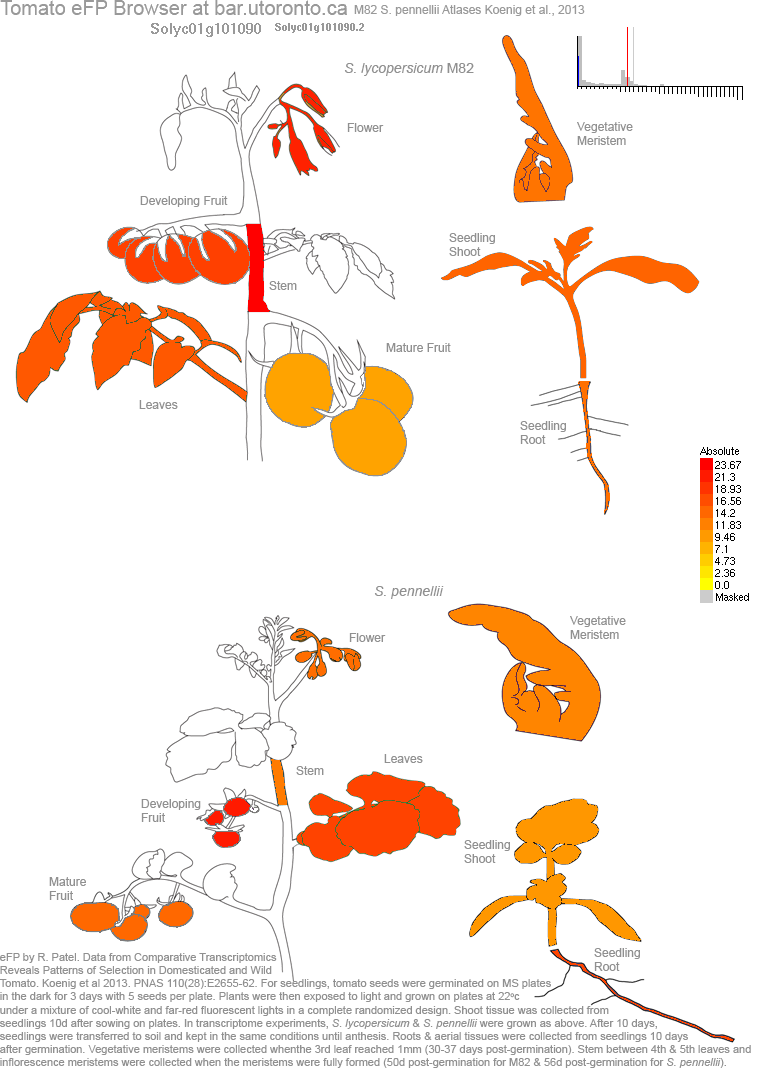
***

***RabGAP20***

***
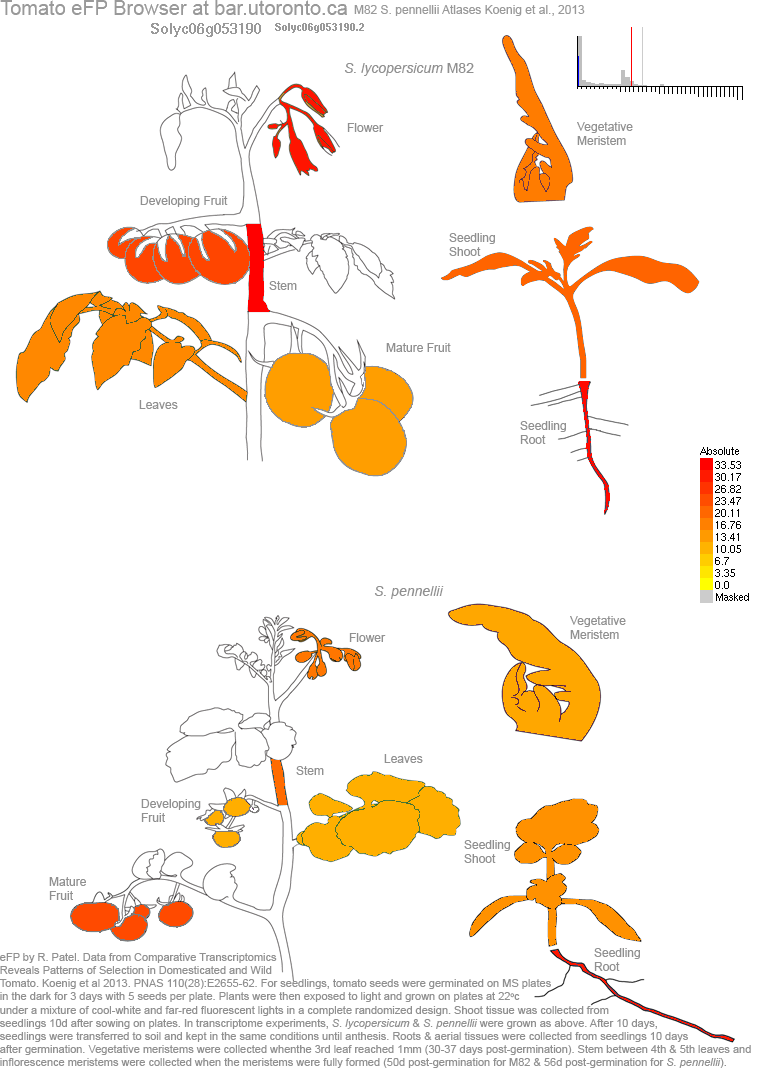
***

***RabGAP21a***

***
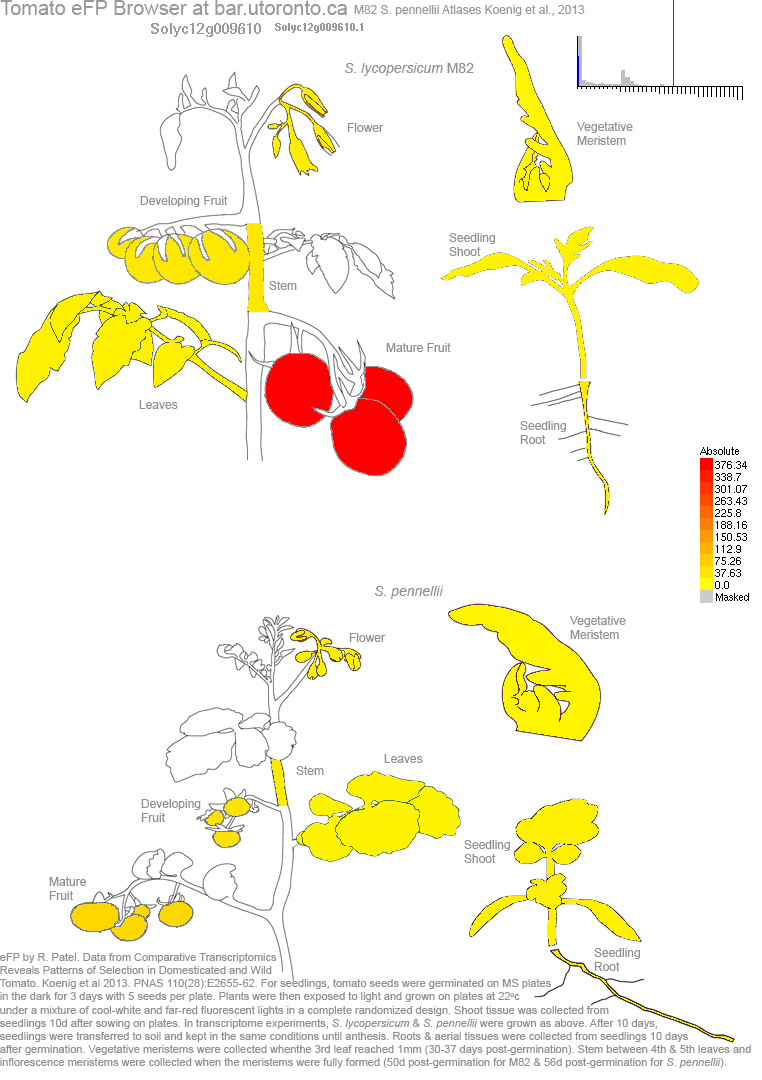
***

***RabGAP21b***

***
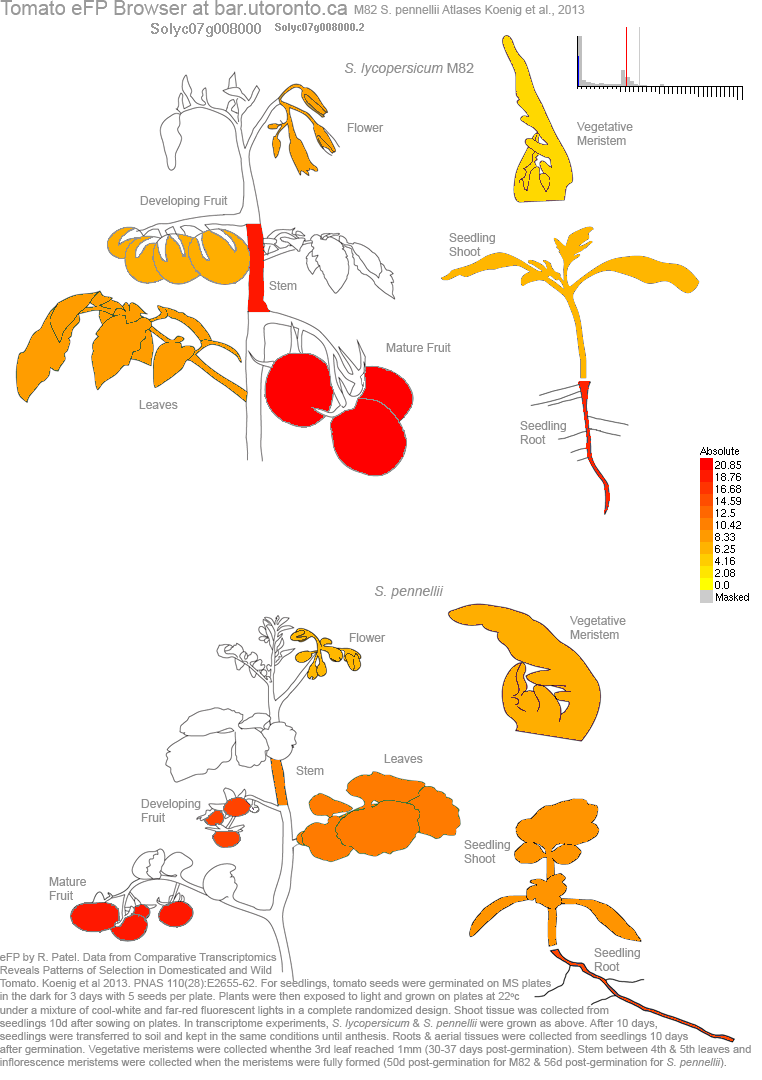
***

***RabGAP22***

***
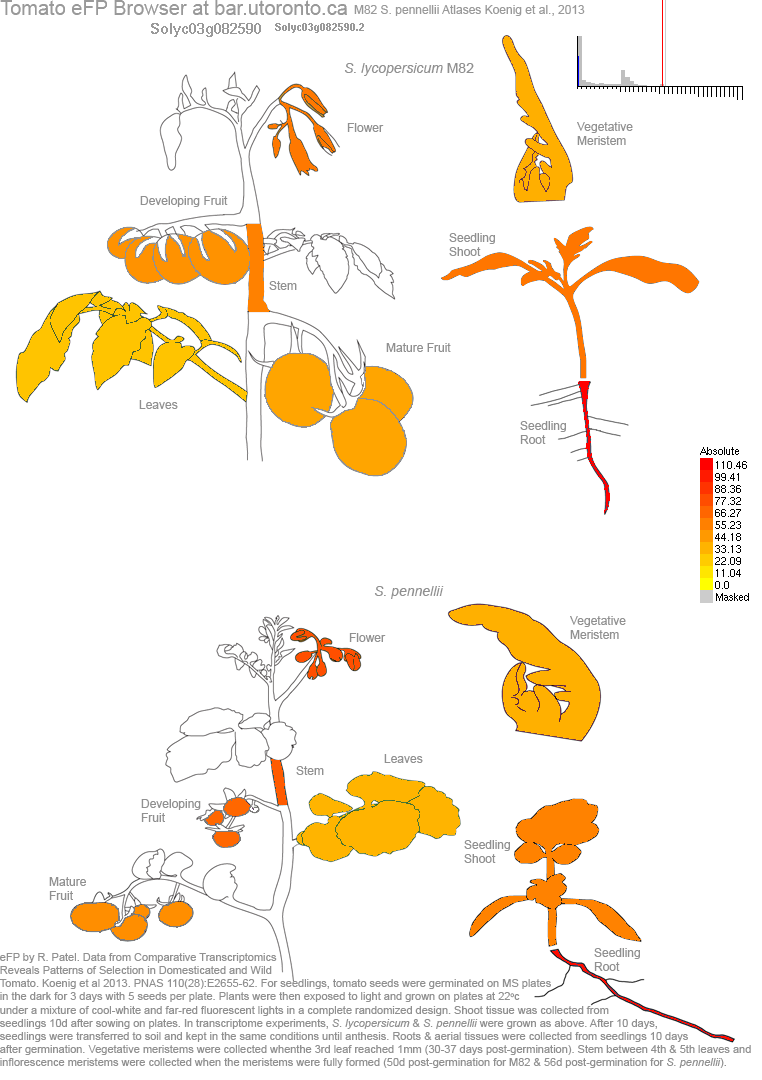
***

***RabGAP23a***

***
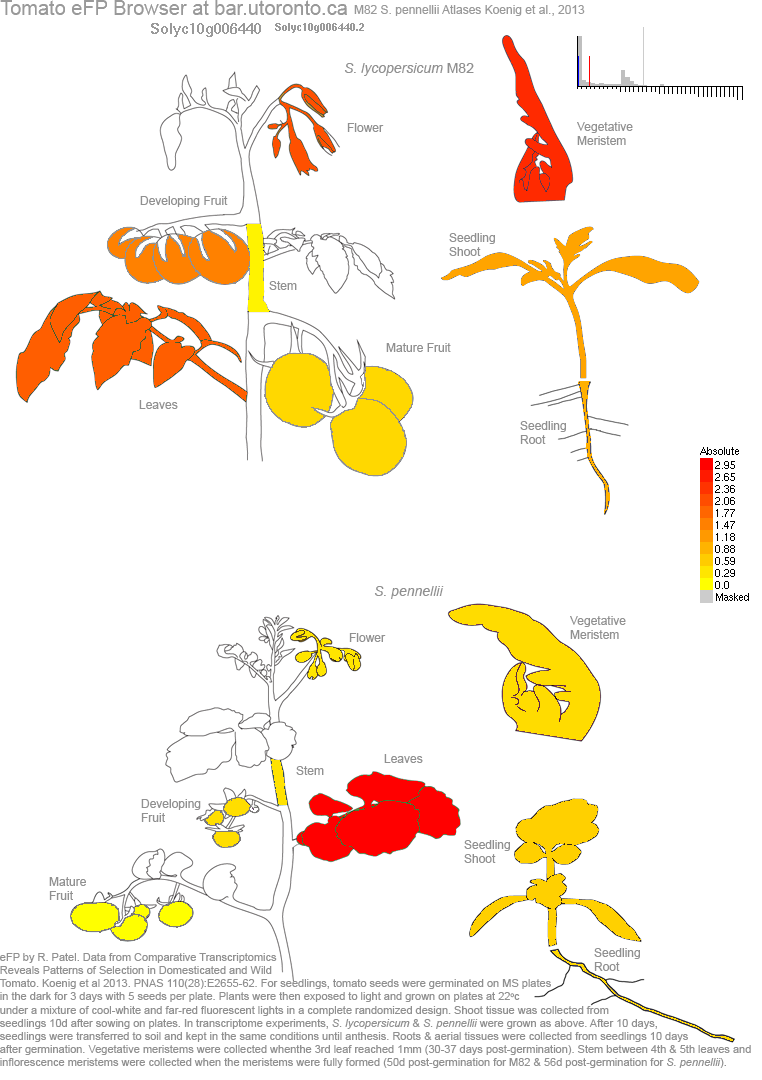
***

***RabGAP23b***

***
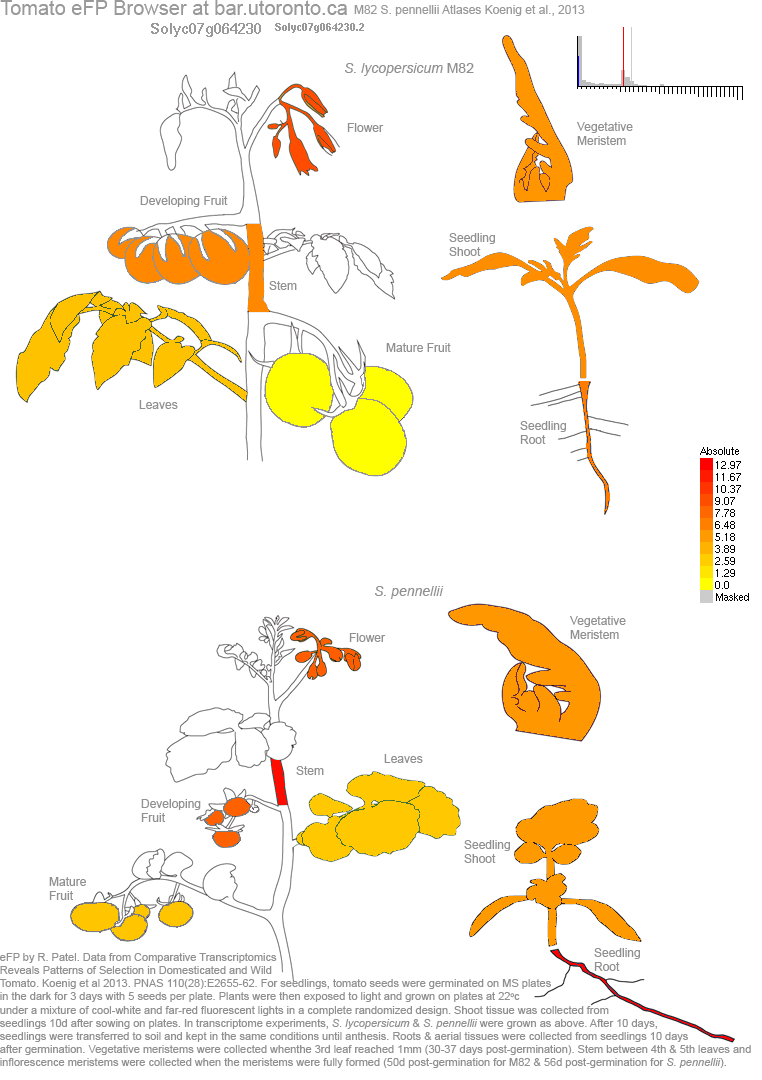
***
